# Extended reality in supporting cancer patients and survivors: A systematic review on the benefits and challenges across the cancer care continuum

**DOI:** 10.1016/j.fhj.2025.100483

**Published:** 2025-10-28

**Authors:** Safa Elkefi, Achraf Tounsi, Siwar Boudiche, Alicia K. Matthews, Rose Hernandez, Noureddine Lourimi

**Affiliations:** a School of Systems Sciences and Industrial Engineering, Watson College of Engineering, Binghamton University, Vestal, NY, USA; b Roadie, Atlanta, GA, USA; c Industrial Engineering Department, National Engineering School of Tunis, Tunis, Tunisia; d School of Nursing, University of Illinois Chicago, Chicago, USA; e National Engineering School of Tunis, Tunis, Tunisia

**Keywords:** Virtual reality, Immersive technology, Augmented reality, Cancer, Treatment, Survival, Diagnosis, Screening

## Abstract

**Purpose:**

Our study explores the role of extended reality (XR) in supporting cancer patients.

**Methods:**

A systematic search was conducted across six electronic databases. We adhered to the guidelines of the Preferred Reporting Items for Systematic Reviews and Meta-Analysis (PRISMA) reviews.

**Results:**

We included 62 studies. XR technologies were mainly applied during the treatment phase (64.5% of studies), with additional applications in palliative care and during survivorship (33.9%). One study addressed the diagnosis phase, and none supported prevention or early detection. XR showed effective results supporting patients in seven areas: anxiety and stress reduction (n = 40), pain management (n = 19) and symptom tracking (n = 14). Challenges identified related to physical discomfort, technical issues, and user compliance.

**Conclusions:**

XR interventions demonstrate significant potential in improving patient outcomes, particularly in anxiety reduction and symptom management. However, their application remains unevenly distributed across the cancer care continuum.

## Highlights

• Extended reality is gaining more and more interest in healthcare applications.
• XR tools have the potential to improve patient outcomes in cancer care.
• XR is commonly used in anxiety and symptoms management.

## Introduction

Cancer is among the leading causes of death worldwide. In 2022, there were approximately 20 million new cancer cases and 9.7 million cancer-related deaths.^1^ By 2040, cancer incidence is expected to rise to 29.9 million and cancer-related deaths to 15.3 million.^1^ A cancer diagnosis and treatment is associated with significant physical, psychological, social and financial challenges.^2^ A meta-analysis conducted by Zhang and colleagues reported that a significant proportion of cancer patients experience depression (32%) and general distress (53.9%) during the active phases of treatment.^3^ In addition, approximately one in four cancer survivors report persistent problems, including anxiety, depression, and other forms of psychological and psychosocial distress.^4^ Further, a review by Smith *et al* found that 49% of patients report a significant financial burden following a cancer diagnosis.^5^ These findings highlight several of the adverse short and long-term sequelae associated with a cancer diagnosis and underscore the need for interventions to improve patient-centred care and support throughout the cancer care continuum.

Health technology tools such as wearable devices, mobile health, telehealth and patient portals facilitate the adoption of patient-centred approaches in cancer care.^6^ They have the potential to improve the delivery of cancer care through enhanced patient–provider communication, symptom assessment and management, and optimised patient engagement across the cancer care continuum.^6^^,^^7^ They have also been used to support the mental and psychological wellbeing of patients, which contributes to improved quality-of-life outcomes.^8^ Additionally, these tools have been shown to support patients across the cancer care continuum, from supporting prevention, early detection efforts, diagnosis and treatment to palliative care and survivorship needs.^9^^,^^10^ With technological advances, new tools have emerged that hold promise for improving support for cancer patients. Several emerging technologies in the field of cancer care are based on extended reality techniques (XR).

Extended reality encapsulates a range of tools that blend physical and virtual environments and are available in three formats: virtual (VR), augmented (AR) and mixed (MR) reality.^11^ Virtual reality (VR) utilises computer modelling and simulation to create a fully immersive digital environment. Augmented reality (AR) overlays digital elements in the real world, enhancing the perception of the environment by creating a composite view. Mixed reality (MR) integrates virtual and physical worlds, enabling interaction between both realms.^12^ Over the last decade, increased efforts have been made to integrate these technologies into healthcare, motivated by their scalability and cost-saving benefits.^13^ Different studies have supported the role that immersive technology has in reducing cancer-related anxiety symptoms, improving treatment adherence, and increasing satisfaction with the care received.^14^^,^^15^

Many reviews investigated the potential of XR in supporting patients in their care continuum in non-cancer-related settings.^16^ For instance, studies investigated the use of XR in mental health,^17^ ophthalmology,^18^ neurology,^19^ cardiology,^20^ and other settings to support patient outcomes. However, little attention has been given to investigating applications supporting cancer care outcomes. A study by Sansoni *et al* explored the XR-based interventions that help support the psychological wellbeing of cancer patients.^21^ Many other reviews explored the potential of these technologies in supporting oncologists’ and healthcare teams’ training in cancer care during treatment,^22^, ^23^, ^24^, ^25^ and diagnosis.^26^ Studies supporting patients in different phases of care covered only specific types of cancer, such as breast cancer.^27^

To address the identified gap, this review asks: How are extended reality (XR) technologies applied to support adult cancer patients at each stage of the cancer care continuum, what targets and outcomes have XR-based interventions achieved, and what implementation challenges have been encountered? We then (1) characterise the specific targets of XR interventions, (2) evaluate their reported effectiveness, and (3) summarise practical challenges in their use and implementation.

## Methods

### Search strategy

A systematic search was conducted across six electronic databases: ProQuest, Scopus, PubMed, Web of Science, Embase and IEEE Xplore for all relevant publications from inception to 15 June 2024. The study protocol was published in the Open Science Framework (DOI 10.17605/OSF.IO/DHFZG). The search strategy was developed following PICO (Population, Intervention, Comparison, Outcome) principles, where:

• Population (P): Adult cancer patients (18+ years).
• Intervention (I): Extended Reality (XR) technologies, including Virtual Reality (VR), Augmented Reality (AR) and Mixed Reality (MR).
• Comparison (C): Based on the phases of the cancer care continuum (before, active and post-treatment).
• Outcome (O): Measured effects of XR interventions on patient-related outcomes (eg, anxiety, pain, adherence, quality of life).

**Fig. 1:**
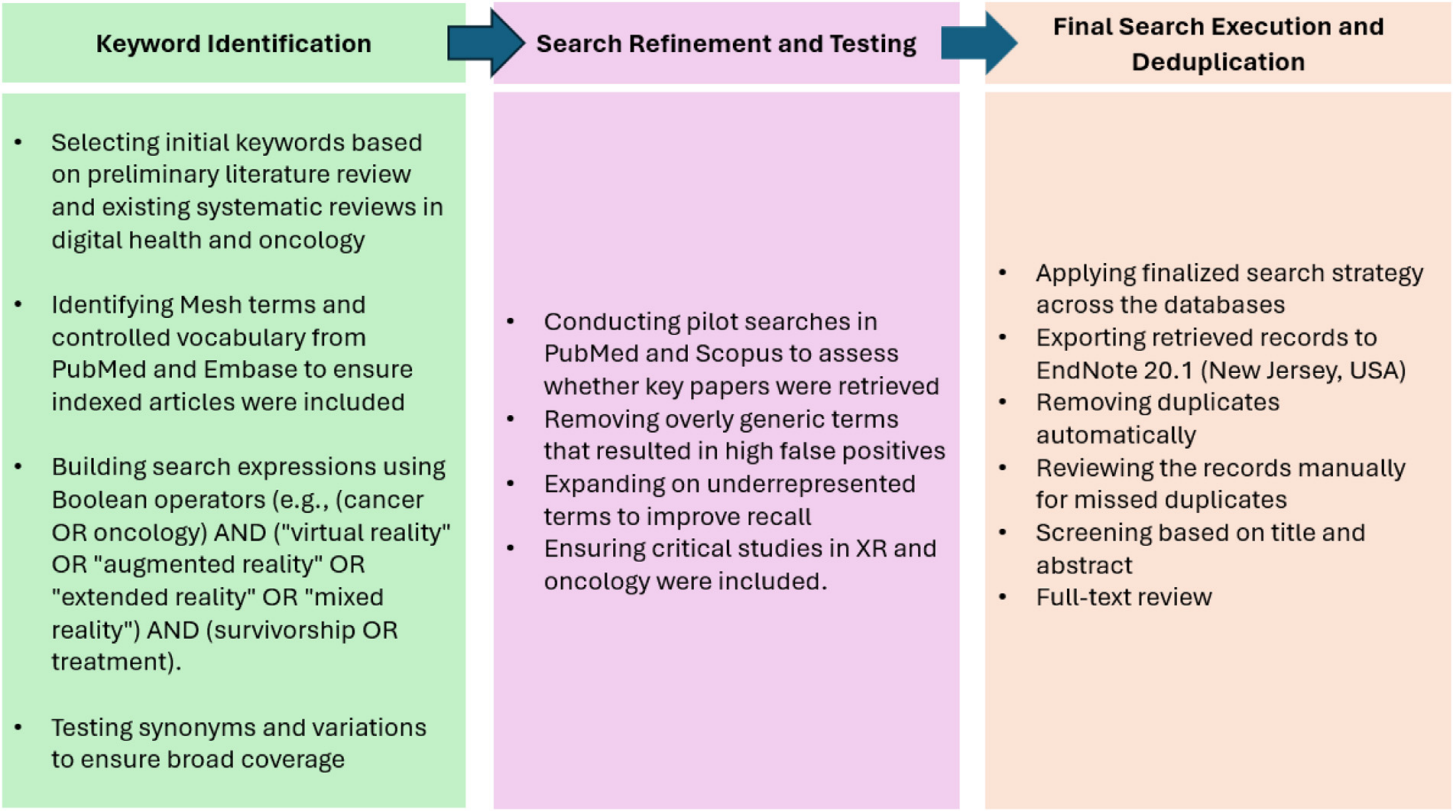
Structured multi-step process of search.

**Fig. 2:**
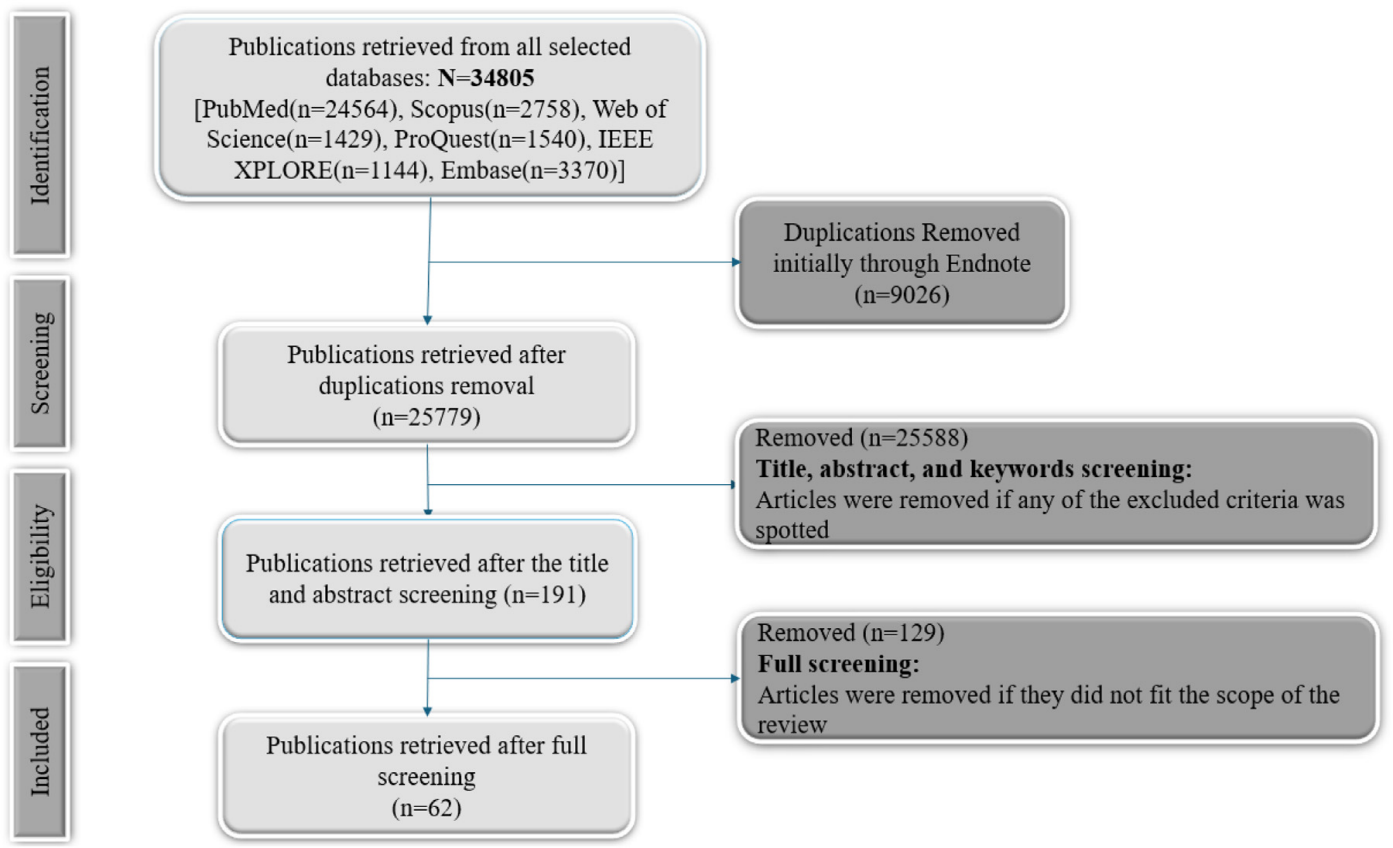
PRISMA flowchart for the search process.

To enhance search comprehensiveness and reproducibility, we followed a structured multi-step process as shown in Figs. 1 and 2.

More detail on the methods (selection process and inclusion criteria) are summarised in Appendix 1. More detail on the risk assessment and its results are described in Appendices 1 and 2.

## Results

**Fig. 3:**
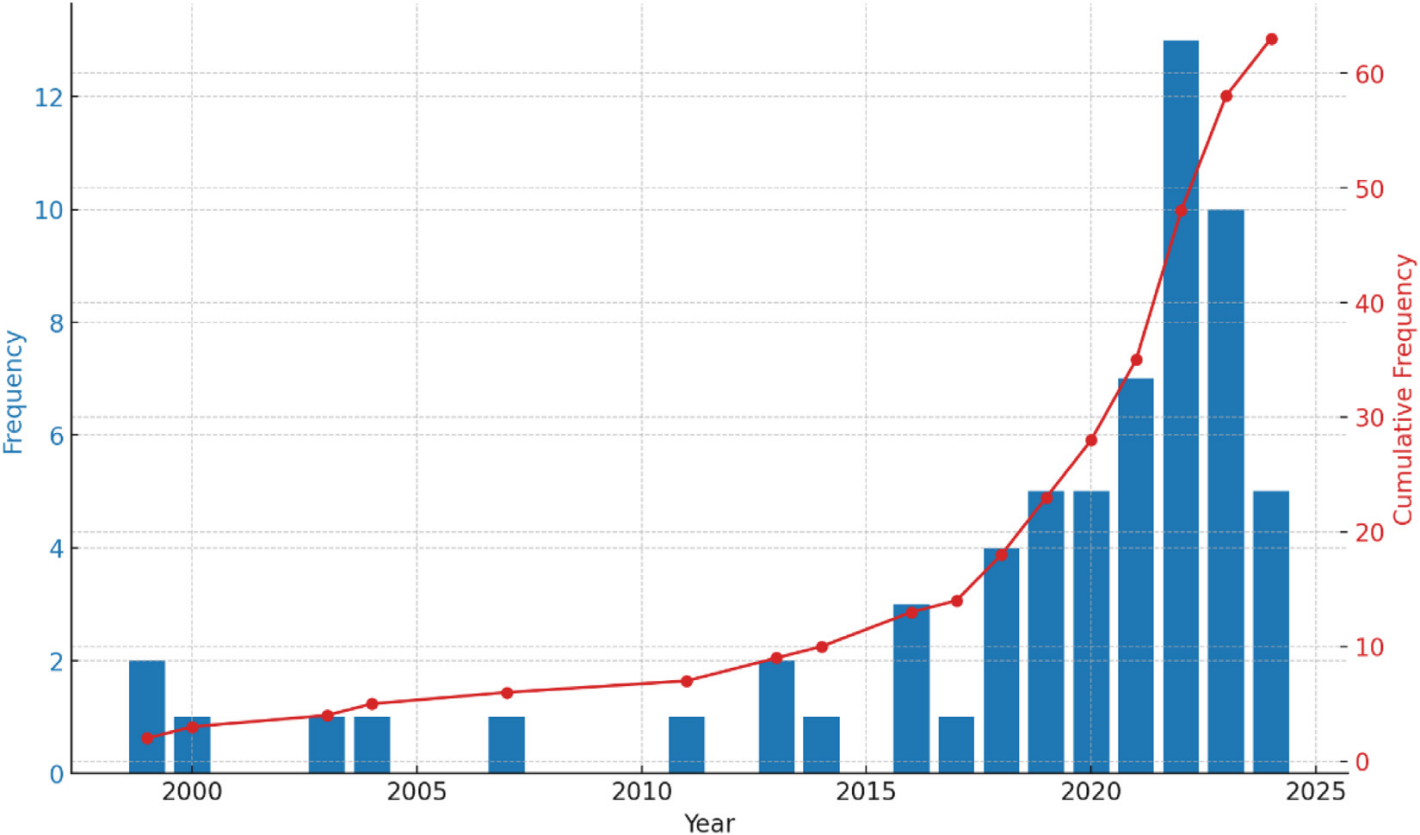
Distribution of the articles over time.

Appendices 3 and 4 describe the 62 included articles and technologies used (see Fig. 3).

**Fig. 4:**
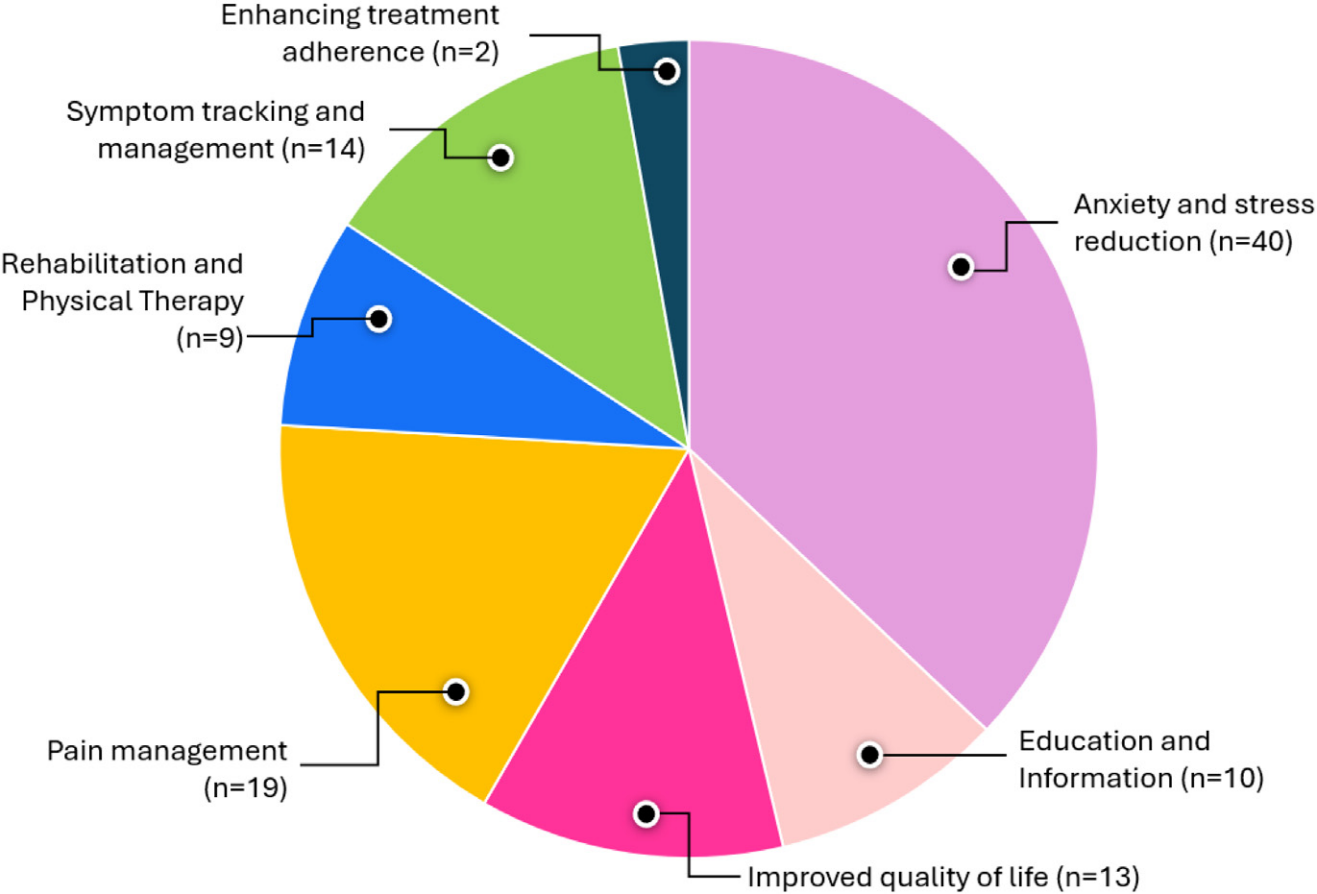
Distribution of the themes identified in the articles.

As shown in Fig. 4, most of the articles reviewed focused on reducing patient anxiety and stress resulting from diagnosis and treatment (n = 40/62). Pain management support was the second most common target of XR interventions (n = 19/62), followed by interventions to help patients with symptom tracking and management and quality of life concerns (n = 14/62). Nine studies focused on rehabilitation and physical therapy support, and n = 10 on increasing patient education and information. Only two studies focused on support for treatment adherence. The outcomes measured used in the included studies are summarised in Appendix 5.

### Anxiety and stress reduction (psychological support)

The studies on anxiety, stress reduction and psychological wellbeing used different VR-based interventions,^28^, ^29^, ^30^, ^31^, ^32^, ^33^, ^34^, ^35^, ^36^, ^37^, ^38^, ^39^, ^40^, ^41^, ^42^ and they covered the cancer care continuum phases of diagnosis, treatment, palliative care and survivorship.

*a. Anxiety and stress reduction during the diagnosis phase*

Patients experience a high level of stress when undergoing diagnosis. King *et al* used VR-delivered relaxation sessions to support patients experiencing distress when undergoing neuroimaging to monitor cancer status.^43^ The VR software loaded on the headset was designed by AppliedVR™ for use within clinical populations and aimed to target unpleasant symptoms and promote relaxation. Qualitative feedback from patients showed high satisfaction with this approach, and all patients adhered to the intervention.^43^

*b. Anxiety and stress reduction during the treatment phase*

#### Preparedness for treatment

VR was used to prepare cancer patients for treatment by helping them adapt to the related stress. For instance, an exergaming protocol (technology-driven physical activities) and VR glasses were used so that patients could watch and listen to the beach and nature.^31^^,^^44^ The intervention was found to result in the reduction of anxiety immediately after the intervention for haematology cancer patients and breast cancer patients.^31^^,^^44^ Another study presented the VR-based mindfulness training model (MTVR) for ovarian cancer patients to prepare them for chemotherapy.^45^ The intervention included four functional modules: personalised curriculum, intelligent monitoring, emotion tracking and Funny Games.^45^ The intervention results in a reduction in anxiety, depression and cancer-related fatigue.^45^ Specifically, the anxiety scores decreased from 5.27 to 2.92, depression scores from 4.96 to 3.13, and cancer fatigue scores from 31.98 to 23.31 after the intervention.^45^ Furthermore, a tool developed by Garrett *et al* provided a realistic virtual environment where patients can experience the various stages of their surgical admission process to be mentally prepared and able to control their stress.^46^ The tool significantly reduced patients’ depression from 7 to 5 (*p* < 0.001), and stress and anxiety were also significantly reduced from 19 to 13 (*p* < 0.001).^46^

Other interventions, VR-based training and resistance exercises, aimed to reduce anticipatory anxiety, depression, and emotional distress in breast cancer patients before their first chemotherapy dose.^47^ The findings were compared to a traditional psychoeducational intervention.^47^ They showed that while both approaches resulted in less anxiety and stress post-intervention, the decrease was more significant in the group that received the VR intervention.^47^ Additionally, at 3 months, the VR group exhibited a more notable decrease in the Emotional Discomfort Detection Scale.^47^ The coping strategies of fighting spirit and cognitive avoidance, assessed with the MINI-MAC coping scale, also increased more in the VR group, with significant differences observed in all evaluations.^47^ Another VR intervention was used to reduce perioperative anxiety in patients undergoing colorectal cancer surgery.^48^ The VR software simulated all perioperative phases that the patient is awake for, from admission to discharge, which resulted in less stress and anxiety compared to regular chemotherapy sessions.^48^

#### Distraction during treatment

Distracting patients using virtual environments (eg, nature-inspired scenarios, movies, etc.) while undergoing treatment was very helpful in supporting the psychological well-being of cancer patients.^36^^,^^38^^,^^48^, ^49^, ^50^, ^51^, ^52^, ^53^, ^54^, ^55^, ^56^, ^57^ For instance, Patient’s Dreams is a ‘distraction therapy’ for use during the first cycle of chemotherapy.^49^^,^^50^ The tool allowed participants to select a nature theme during treatment to shift their mood and promote relaxation. The authors reported a statistically significant reduction in anxiety and stress associated with symptom management during chemotherapy by distracting them with VR.^49^ Breast cancer participants felt more calm (*p* = 0.017), less tense (*p* = 0.042), more relaxed (*p* = 0.002), and more content (*p* = 0.000).^49^ The authors also reported that the tool helped reduce anxiety and alter the perception of time during treatment for patients with different types of cancer,^50^ with positive outcomes that persisted over time, 48 hours after the experiment (state anxiety values reduction by 37.42%).^50^

*c. Anxiety and stress reduction during the palliative care and survivorship phase*

The benefits of immersive technology were also seen through applications that helped patients manage anxiety and stress related to cancer and treatment in the post-treatment phase to help in their transition to the palliative care stage.^42^^,^^58^^,^^59^ Most of these applications used natural environments such as green and blue spaces.^58^ To manage psychological distress resulting from treatment, a study by Reynolds *et al* featured local Florida scenes, including trees, water features, creeks, animals and local parks.^58^ Nature sounds of chirping birds and running water were added to enhance the relaxation effect.^58^ The experiment resulted in more relaxation and less stress resulting from chemotherapy procedures among cancer patients.^58^ Another intervention, entitled SafeSpace, incorporated compassionate mind training (CMT).^60^ The intervention included three short sessions focusing on relaxation and self-compassion exercises.^60^ Mental wellbeing improved significantly after each session (VR 1 = 2.846; VR 2 = 2.501; VR 3 = 2.492). Significant reduction in stress levels from baseline to post-session 3 (z = −2.138, *p* = 0.03).^60^

For stress resulting from cancer symptoms, applications such as VR Blue and Bubble were shown to result in significant improvements. VR Blue used a calming underwater environment and helped decrease anxiety by 65%, improve mood by 70%, and decrease stress by 68%.^61^ Bubble, on the other hand, was used for psychological wellbeing associated with cancer symptoms, including stress, anxiety and sleep disturbances in women with breast and ovarian cancer.^30^ The intervention incorporated cognitive behavioural therapy and mindfulness-based stress reduction elements, delivered in a home-based VR environment designed to provide a calming and cooling experience, which reduced stress by 17.18% and psychological distress by 20.49%.^30^

Combining VR with AI (VR-AI), Horesh *et al* were able to reduce treatment and cancer-related pain, resulting in less anxiety among patients. While immersing in ‘Ocean Rift’, a deep-sea diving experience, or ‘Happy Place’, a tranquil beach setting, breast and ovarian cancer patients experienced less anxiety (from 50.13 to 37.68).^62^ Another application that studies used was Yuma’s World. It showed no changes in anxiety levels when used as a complementary therapy in-home palliative care among breast and ovarian cancer patients.^29^ However, using a basic three-level skill game in a calm underwater environment helped support patients with rectal cancer.^59^ This intervention resulted in significantly reduced anxiety (*p* = 0.042) and tiredness (*p* = 0.001).^59^ Furthermore, the Bedside Wellness System, a virtual forest walk system designed to improve the emotional wellbeing of bedridden patients with lung cancer, helped reduce stress, and relaxation with persistent results after 48 hours of the experiments.^63^ The system used a wide three-screen LCD with stereo sound, a walking system for the bedridden, a scent system with a gentle breeze, and a system for monitoring vital signs. It offered different scenic scenarios, such as a park, a plateau, or a cherry blossom avenue.^63^ Another interesting application of VR was the usage of Google Earth VR to simulate travel experiences for terminal cancer patients, aiming to fulfil their desire to visit a memorable place.^64^ It was able to help in reducing depression (*p* = 0.001), anxiety (*p* < 0.001) and wellbeing (*p* < 0.001).^64^

Finally, the virtual smash room developed by Persson *et al* aimed to vent frustration among cancer patients.^65^ The users can move around within a limited area and interact with objects in the virtual environment using hand controllers.^65^ They can pick up breakable objects and throw them or smash them against surfaces in the room.^65^ There are also virtual tools the users can pick up and use to hit the breakable objects, such as a wooden paddle, a morning star, a hammer or a crowbar.^65^ This exercise requires the user to be physically active by moving around and waving their arms.^65^ The users can interact with the system by standing up or sitting down, depending on the individual’s physical limitations.^65^ The intervention helped retain people’s focus (mean 4.44, maximum 5, SD 0.74).^65^

### Pain and symptom tracking and management

Through the use of extended reality applications, the included studies showed important improvements in outcomes related to symptom management with a focus on symptoms such as pain, hot flashes, sleep disturbance, tiredness, shortness of breath, drowsiness, nausea and vomiting.^28^^,^^30^^,^^36^^,^^38^^,^^49^^,^^55^^,^^63^^,^^64^^,^^66^, ^67^, ^68^

*a. Pain and symptom tracking and management during the treatment phase*

Turrado *et al* developed a tool that teaches patients how to manage their symptoms before colorectal cancer surgery.^36^ The VR software recreated all perioperative phases in which the patient is awake, from admission to discharge.^36^ Another tool by Gao *et al* was developed to educate patients on the radiotherapy processes and help them learn more about symptom tracking.^38^ The interventions showed that patients experienced less anxiety and a significant decrease in systolic blood pressure (*p* < 0.05).^38^ VR has also been demonstrated to be effective in assisting cancer patients in symptom management at home and after treatment sessions.^67^ Giannelli *et al* used Virtual Reality Intervention Therapy (VRIT) to treat anticipatory nausea and vomiting in cancer patients undergoing chemotherapy.^67^ The therapy utilises a head-mounted display (HMD) system to immerse patients in a virtual environment, providing a distraction from the negative anticipatory symptoms associated with chemotherapy. Symptoms such as pain, tiredness, nausea and vomiting were monitored. The study found that VRIT effectively reduced nausea and vomiting.^67^ Specifically, 80% of the participants reported a decrease in the level of nausea and the number of vomiting episodes.^67^ The interventions also showed a significant improvement in pain (*p* = 0.013), tiredness (*p* < 0.001) and anxiety (*p* = 0.013).^67^ Notably, more focus was given to pain and physical wellbeing as symptoms**.**^69^^,^^70^ For instance, using the calming underwater environment, the Blue tool, helped reduce patients’ pain by 59%.^61^

Another one by Feyzioğlu *et al* discussed a VR-delivered pain therapy software program designed to help cancer patients undergoing breast cancer surgery manage neuropathic pain.^71^ The program incorporated guided visualisation and progressive muscle relaxation techniques to minimise cybersickness in this vulnerable patient group.^71^ It helped reduce opioid consumption and pain severity among breast cancer patients.^71^ At 1-month and 3-month follow-ups, the intervention group showed a trend towards reduced opioid consumption compared to the control group. At 1 month, the reduction was 8 mg in the intervention group versus no change in the control group (*p* = 0.52).^71^ At 3 months, the reduction was 4 mg in the intervention group versus an increase of 15 mg in the control group (*p* = 0.34).^71^ The intervention group also showed a trend towards reduced pain severity. At 1 month, the reduction was 0.4 in the intervention group versus an increase of 0.4 in the control group (*p* = 0.02).^71^

*b. Pain and symptom tracking and management during the palliative care and survivorship phase*

XR tools were also developed to support symptom tracking and management in the post-treatment phase. For instance, Bubble was used to manage symptoms such as hot flashes and sleep disturbances in women with breast and ovarian cancer.^30^ The intervention incorporated cognitive behavioural therapy and mindfulness-based stress reduction elements, delivered in a VR environment designed to provide a calming and cooling experience.^30^ It helped reduce the daily frequency of hot flashes from pre-treatment M = 11.31 to post-treatment M = 6.83, *p* < 0.01.^30^

Additionally, UNICARE Home+ showed significant outcomes improvement.^72^ The tool is an AR application that helps manage pain in rehabilitation therapies after treatment and allows remote monitoring by therapists while breast cancer patients are at home.^72^

Furthermore, Mohammad *et al* used VR therapies as an adjunctive treatment for managing chronic cancer pain, using distracting techniques to help breast patients adapt to the side effects of chemotherapy.^73^ A study by Atef *et al* discussed the use of 3D head-mounted and 2D screen VR applications to manage cancer pain in adults receiving palliative care.^74^ Participants showed a reduced pain intensity of 1.9 (*p* = 0.003) for the 3D HMD VR and a reduction of 1.5 (*p* = 0.007) for the 2D screen applications.^74^

Using virtual travel sessions to memorable or desired locations, Cartujano-Barrera *et al* helped decrease pain from a mean score of 2.35 to 1.15 (*p* = 0.005), tiredness from a mean score of 2.90 to 1.35 (*p* = 0.004), drowsiness from a mean score of 2.70 tot 1.35 (*p* = 0.012), and shortness of breath from a mean score of 1.74 to 0.35 (*p* = 0.022).^66^

### Rehabilitation, physical therapy and improved quality of life

Different tools were developed to support rehabilitation and physical therapy among cancer patients, contributing to the improvement of their quality of life.^72^^,^^75^^,^^76^

*a. Rehabilitation, physical therapy, and improved quality of life during the treatment phase*

Chuan *et al* developed a bespoke VR-delivered pain therapy to help cancer patients manage neuropathic pain, incorporating guided visualisation and progressive muscle relaxation techniques while minimising the risk of cybersickness in this vulnerable patient population. The intervention showed improved pain severity and quality of life.^75^ Moreover, Feyzioğlu *et al* used a Kinect-based VR rehabilitation program to improve upper limb function in patients undergoing breast cancer surgery.^71^ The intervention improved grip strength, functionality, muscle strength, and shoulder range of motion (ROM).^71^

*b. Rehabilitation, physical therapy, and improved quality of life during the palliative care and survivorship phase*

UNICARE Home+, an AR-based digital healthcare system for postoperative rehabilitation among breast cancer patients, provides at-home exercises.^72^^,^^77^ It uses the Xbox One Kinect to track movements in three-dimensional space, providing real-time feedback through visual and auditory cues to ensure exercises are performed correctly.^72^^,^^77^ The system delivers prescribed exercises directly to the patient’s home, where they can follow along with on-screen instructions and receive instant feedback.^72^^,^^77^ This setup allows physicians to remotely monitor and customise the exercise programme, enhancing the convenience and effectiveness of rehabilitation without the need for frequent hospital visits.^72^^,^^77^ The system aims to improve shoulder range of motion, reduce pain, and enhance functional outcomes and quality of life for postoperative breast cancer patients.^72^^,^^77^ With this setup, the studies improved outcomes such as ROM and functional outcomes over 12 weeks, all while improving the quality of life among the participants.^72^^,^^77^ Another study by Basha *et al* used Xbox Kinect games to provide complex decongestive physiotherapy, including manual lymphatic drainage, compression therapy and exercises.^47^ Compared to traditional therapy, statistically significant differences were recorded in VAS (pain intensity), Disability of the Arm, Shoulder, and Hand (DASH), shoulder ROM (*p* < 0.001), bodily pain (*p* = 0.002), general health (*p* < 0.001), and vitality (*p* = 0.006) in favour of the VR group.^47^

### Enhancing treatment adherence

Two interventions aimed to improve the patient’s adherence to cancer treatment by providing mindfulness exercises during treatment sessions.^41^^,^^78^ Schrempf *et al’s* intervention involved patients chairside in a hospital bed depending on the patient’s condition and preference,^78^ and provided them with calming effects to encourage ongoing treatment.^78^ While Schrempf *et al* supported patients during the treatment phase, Banos *et al’s* intervention helped improve adherence to post-treatment exercise among people with advanced cancer by improving their mood and wellbeing.^41^

### Education and information

*a. Education and information during the treatment phase*

VR was used to provide cognitive behavioural therapy to help patients manage their depressive symptoms during the treatment sessions.^34^^,^^79^^,^^80^ The findings from the study by Cimpean *et al* suggested significant improvements in quality of life and pain intensity, *p* < 0.05,^34^ and Fabi *et al* showed better anxiety levels.^79^ Another intervention by Garrett *et al* helped patients manage chronic cancer pain through distraction, relaxation and cognitive engagement. It educated them by integrating mindfulness meditation techniques and providing guided experiences that enhanced their understanding and acceptance of their pain.^46^

*b. Education and information during the palliative care and survivorship phase*

Educating patients on the benefits of training exercises on symptom severity and quality of life through exercise sessions.^47^ A study by Zhou *et al* was effective in teaching patients about the benefits of functional exercise and how to perform physical rehabilitation results.^81^ Moscato *et al* helped teach patients about their post-treatment symptomology from the comfort of their homes, resulting in better pain and anxiety levels.^68^ Niki *et al* also educated patients on how to cope with terminal cancer by allowing them to virtually ‘go to a memorable place’ or ‘return home’.^60^

### Challenges of extended reality use

The studies and interventions highlighted several challenges worth noting as part of this review. The challenges involved physical discomfort and health concerns, technical and operational challenges, environmental and contextual challenges, resource and cost challenges, and user experience and compliance. More details on these challenges are in Appendix 6.

## Discussion

This review investigated the different applications of extended reality (XR) technologies to support cancer patients across the cancer care continuum. It explored their impact on patient outcomes in the different phases (diagnosis, treatment, survivorship and palliative care) and described their use challenges. Sixty-two studies were included, suggesting the extent of literature on cancer support using XR. Although a review by Zhu *et al* showed that AR is gaining more popularity in healthcare,^82^ only two studies in our review showed results from AR-based experiments to support cancer patients. Notably, most of the studies included in the review by Zhu *et al* presented early prototypes only,^82^ which may explain the limited use of this technology in cancer settings. The included studies have also shown support for different types of cancer patients, with more focus on technologies developed for general cancer populations (more than two types of cancer) and breast cancer patients. While focusing on the general cancer population may be beneficial to different patients, it can result in less customised experiences and less efficient outcomes.^83^ More focus should also be given to different types of cancer, such as lung and gastrointestinal cancer populations, where technology-based educational interventions are needed to support their behaviours and decision-making.^84^

### Technology functions of support to cancer patients across the cancer care continuum

Furthermore, this study identified the different functions of the XR technologies available to support cancer patients. The functions were categorised into seven groups, ranked according to the predominance of the targeted health outcome: anxiety and stress reduction, pain management, symptom tracking and management, improved quality of life, rehabilitation and physical therapy, education and information, and enhancing treatment adherence. While many reviews investigated the use of immersive technology to support psychological wellbeing and rehabilitation management of specific groups of cancer patients,^21^^,^^85^ our study, to our knowledge, adds to the literature by identifying these different themes, showing that not only do these technologies have different potential applications for all the different groups of cancer patients, but some applications are also given more attention than others. For instance, while enhancing treatment adherence may be very important to cancer patients, more attention is given to psychological wellbeing support.^86^ It is important to note that improving treatment adherence can also result in better psychological outcomes.^87^ Our results emphasised the distribution of these different functions across the cancer care continuum. We found a predominant focus on the treatment phase, followed by the palliative care and survivorship phase. The limited exploration in prevention, early detection, and diagnosis underscores significant research gaps. Clinicians and researchers should prioritise studies investigating XR technologies’ potential in these early stages of cancer care. Supporting cancer patients in the early stages can help enhance patient engagement, adherence to treatment protocols, and overall quality of life,^2^^,^^88^ and exploring how XR can enhance early diagnosis or facilitate preventative strategies may lead to earlier interventions and improved patient outcomes, considering the role played by other medical informatics tools.^89^

### Challenges of extended reality use

When investigating the challenges accompanying the use of XR in cancer settings, patients mentioned several problems they faced related to their physical discomfort and health concerns, technical and operational challenges, environmental, resource, cost, and contextual challenges, and user experience problems. While most of the studies ensured that these challenges did not impact the outcomes of the studies, it is essential to address them and account for them when designing future experiments to improve cancer patients’ acceptability of these technologies. Some technical and operational challenges can be solved to provide cancer patients with better testing environments and experiences. For instance, extensive literature focuses on improving cybersickness issues in XR experiments by adjusting the settings to the levels of comfort among patients, allowing patients to take breaks between sessions, and using visual techniques such as rotation blurring, among others.^90^, ^91^, ^92^

While many of these techniques have been tested in general care settings,^90^^,^^92^ more attention should be given to testing them in cancer care settings, considering cancer patients may suffer from disease-related side effects that could contribute to more pronounced technology use issues. While many studies used commercial software in their experiments, simply trying commercially available products on different cancer populations might not suffice and may be harmful. Some challenges related to physical discomfort could also be mitigated by providing more ergonomic equipment and adjustable testing environments that may result in less pain. In addition, reducing external noise and disturbances in experimental environments through better soundproofing and controlled settings can result in more user satisfaction. While home interventions can be beneficial in supporting self-efficacy among patients, more structured home interventions with better support should be designed to control the experiments’ outcomes.

Although some challenges can be addressed, others that are related to the physical and psychological health status of the patients are harder to account for. For instance, in some experiments, participants experienced the progression of their diseases, leading to the withdrawal of consent during VR exercises.^92^ This challenge is inherent to the participants’ health conditions and cannot be mitigated through VR design or intervention adjustments.

The study limitations are summarised in Appendix 7.

### Study limitations

While this review has many strengths, it has some limitations worth acknowledging. First, we only included articles written in English. We may have missed relevant articles that are written in different languages. In addition, the XR technologies and interventions included in the study varied widely in terms of content, strategies and specifications. This heterogeneity may complicate the interpretation of results and limit the ability to draw consistent conclusions about the effectiveness of XR technologies across different cancer care settings. Furthermore, the studies reviewed were conducted in various settings (clinic vs. home), and the technologies were tailored to different patient needs. This diversity may limit the generalisability of the findings to other contexts or patient groups, particularly where resources or access to technology are limited. We did not differentiate between research-developed tools and commercially available ones in the reporting of the findings, which can be covered in future research. Finally, this study focused on studying the use of XR across the different cancer continuum phases. Although we reported the type of cancer for each of the populations that the tools were designed for, it would be beneficial to compare the findings per type of cancer in future studies.

Based on the findings, we propose the following key directions for future research. First, a more targeted review examining XR interventions for specific cancer types (eg, breast, lung or haematological malignancies) could provide deeper insight into cancer-specific symptom management and patient needs. Second, the majority of current XR interventions focus on treatment and survivorship. Future research should explore applications in: (1) prevention and early detection (eg, XR-based education and risk assessment tools), and (2) diagnosis (eg, XR-assisted imaging interpretation and biopsy simulations). Additionally, future studies should develop consistent intervention protocols, including: session length and frequency of XR use, optimal headset types and settings for different patient needs, and longitudinal follow-up studies to assess sustained impact over time.

By addressing these research gaps, future studies can provide stronger evidence on the role of XR in oncology, ultimately improving patient outcomes and clinical implementation strategies.

## Conclusions

While XR technologies represent a promising frontier in cancer care, more targeted research and development efforts are needed to realise their full potential. By addressing the identified gaps and challenges, future studies can contribute to the evolution of patient-centred cancer care, offering enhanced support, and improved outcomes for cancer patients throughout their journey.

**Summary table:**

• Extended reality is emerging in healthcare.
• Virtual reality and augmented reality are gaining more interest in cancer care for patients’ use.
• While these technologies have shown promise, there remains a significant gap in their application during the early stages of cancer care, such as prevention, early detection and diagnosis.
• The variability in XR technologies and intervention designs, along with the challenges related to physical discomfort, technical issues, and user compliance, highlights the importance of addressing these limitations to ensure the broader applicability and acceptance of XR interventions in real-world clinical settings.

## Funding

This project was funded by the HICCC at Columbia University under the VELOCITY mechanism for 2024 and 2025.

## CRediT authorship contribution statement

**Safa Elkefi:** Writing – review & editing, Writing – original draft, Visualization, Validation, Supervision, Software, Resources, Project administration, Methodology, Investigation, Funding acquisition, Formal analysis, Data curation, Conceptualization. **Achraf Tounsi:** Methodology, Formal analysis, Data curation. **Siwar Boudiche:** Formal analysis, Data curation. **Alicia K. Matthews:** Writing – review & editing, Validation, Funding acquisition. **Rose Hernandez:** Writing – review & editing. **Noureddine Lourimi:** Formal analysis.

## Declaration of competing interest

The authors declare that they have no known competing financial interests or personal relationships that could have appeared to influence the work reported in this paper.

## Appendix Supplementary materials

Image, application 1

Image, application 2

## References

[1] International Agency for Research on Cancer I. Statistics at a glance: the burden of cancer worldwide. 2022. https://www.who.int/news/item/01-02-2024-global-cancer-burden-growing–amidst-mounting-need-for-services#:∼:text=The%20World%20Health%20Organization%20(WHO)%20released%20estimates,cancer%20cases%20*%2077%25%20increase%20from%202022. Accessed 12 May 2025.

[2] Mao J.J., Pillai G.G., Andrade C.J. (2022). Integrative oncology: addressing the global challenges of cancer prevention and treatment. CA: Cancer J Clin.

[3] Zhang L., Liu X., Tong F. (2022). The prevalence of psychological disorders among cancer patients during the COVID-19 pandemic: a meta-analysis. Psycho-Oncol.

[4] Institute NC. Meeting cancer survivors' psychosocial health needs: a conversation with Dr. Patricia Ganz. https://www.cancer.gov/news-events/cancer-currents-blog/2022/psychosocial-cancer-survivors-patricia-ganz. Accessed June 6, 2024.

[5] Yu H., Li H., Zuo T. (2022). Financial toxicity and psychological distress in adults with cancer: a treatment-based analysis. Asia Pac J Oncol Nurs.

[6] Penedo F.J., Oswald L.B., Kronenfeld J.P., Garcia S.F., Cella D., Yanez B. (2020). The increasing value of eHealth in the delivery of patient-centred cancer care. Lancet Oncol.

[7] ElKefi S., Asan O. (2021). How technology impacts communication between cancer patients and their health care providers: a systematic literature review. Int J Med Informat.

[8] Elkefi S., Trapani D., Ryan S. (2023). The role of digital health in supporting cancer patients' mental health and psychological well-being for a better quality of life: a systematic literature review. Int J Med Informat.

[9] Granda-Cameron C., Kates J., Wen K-Y (2024). mHealth interventions to improve the breast cancer continuum of care from prevention to survivorship of Hispanic women: a scoping review. J Racial Ethn Health Disparities.

[10] Briggs L.G., Labban M., Alkhatib K., Nguyen D-D, Cole A.P., Trinh Q-D (2022). Digital technologies in cancer care: a review from the clinician's perspective. J Comp Eff Res.

[11] Lee H-G, Chung S., Lee W-H (2013). Presence in virtual golf simulators: The effects of presence on perceived enjoyment, perceived value, and behavioral intention. New Media Soc.

[12] Tramosa L. Beyond AR vs. VR: what is the difference between AR vs. MR vs. VR vs. XR? https://www.interaction-design.org/literature/article/beyond-ar-vs-vr-what-is-the-difference-between-ar-vs-mr-vs-vr-vs-xr?srsltid=AfmBOopJTcxK0SJFJ7zr4_xAe6YBExSvrinusDA6u3RMCVcxqqQHdKH1. Accessed June 7, 2024.

[13] Suh A., Prophet J. (2018). The state of immersive technology research: a literature analysis. Comput Hum Behav.

[14] Pittara M., Matsangidou M., Stylianides K., Petkov N., Pattichis C.S. (2020). Virtual reality for pain management in cancer: a comprehensive review. IEEE Access.

[15] Buche H., Michel A., Blanc N. (2022). Use of virtual reality in oncology: from the state of the art to an integrative model. Front Virtual Real.

[16] Logeswaran A., Munsch C., Chong Y.J., Ralph N., McCrossnan J. (2021). The role of extended reality technology in healthcare education: towards a learner-centred approach. Future Healthc J.

[17] Stone J. (2020). The video game debate 2.

[18] Ong C.W., Tan M.C.J., Lam M., Koh V.T.C. (2021). Applications of extended reality in ophthalmology: systematic review. J Med Internet Res.

[19] Dadario N.B., Quinoa T., Khatri D., Boockvar J., Langer D., D'Amico R.S. (2021). Examining the benefits of extended reality in neurosurgery: a systematic review. J Clin Neurosci.

[20] Jung C., Wolff G., Wernly B. (2022). Virtual and augmented reality in cardiovascular care: state-of-the-art and future perspectives. Cardiovasc Imaging.

[21] Sansoni M., Malighetti C., Riva G. (2022).

[22] Zhang J., Lu V., Khanduja V. (2023). The impact of extended reality on surgery: a scoping review. Int Orthopaed.

[23] Checcucci E., Piana A., Volpi G. (2024). Visual extended reality tools in image-guided surgery in urology: a systematic review. Eur J Nucl Med Mol Imaging.

[24] Rutkowski S., Czech O., Wrzeciono A., Kiper P., Szczepańska-Gieracha J., Malicka I. (2021). Virtual reality as a chemotherapy support in treatment of anxiety and fatigue in patients with cancer: a systematic review and meta-analysis and future research directions. Complement Therap Med.

[25] Zasadzka E., Pieczyńska A., Trzmiel T., Hojan K. (2021). Virtual reality as a promising tool supporting oncological treatment in breast cancer. Int J Environ Res Public Health.

[26] Kukla P., Maciejewska K., Strojna I., Zapał M., Zwierzchowski G., Bąk B. (2023). Extended reality in diagnostic imaging—a literature review. Tomography.

[27] Yazdipour A.B., Saeedi S., Bostan H., Masoorian H., Sajjadi H., Ghazisaeedi M. (2023). Opportunities and challenges of virtual reality-based interventions for patients with breast cancer: a systematic review. BMC Med Inform Decis Mak.

[28] Schneider S.M., Kisby C.K., Flint E.P. (2011). Effect of virtual reality on time perception in patients receiving chemotherapy. Support Care Cancer.

[29] Oyama H., Ohsuga M., Tatsuno Y., Katsumata N. (1999). Evaluation of the psycho-oncological effectiveness of the bedside wellness system. CyberPsychol Behav.

[30] Oyama H., Kaneda M., Katsumata N., Akechi T., Ohsuga M. (2000). Using the bedside wellness system during chemotherapy decreases fatigue and emesis in cancer patients. J Med Syst.

[31] Tsuda K., Sudo K., Goto G. (2016). A feasibility study of virtual reality exercise in elderly patients with hematologic malignancies receiving chemotherapy. Internal Med.

[32] da Silva Alves R., Iunes D.H., de Carvalho J.M. (2018). Effects of exergaming on quality of life in cancer patients. Games Health J.

[33] Jimenez Y.A., Cumming S., Wang W., Stuart K., Thwaites D.I., Lewis S.J. (2018). Patient education using virtual reality increases knowledge and positive experience for breast cancer patients undergoing radiation therapy. Support Care Cancer.

[34] Cîmpean A.I. (2019). A pilot study to compare cognitive behavioral therapy with virtual reality vs. standard cognitive behavioral therapy for patients who suffer from cervical cancer. J Evid-Based Psychother.

[35] Chirico A., Maiorano P., Indovina P. (2020). Virtual reality and music therapy as distraction interventions to alleviate anxiety and improve mood states in breast cancer patients during chemotherapy. J Cell Physiol.

[36] Turrado V., Guzmán Y., Jiménez-Lillo J. (2021). Exposure to virtual reality as a tool to reduce peri-operative anxiety in patients undergoing colorectal cancer surgery: a single-center prospective randomized clinical trial. Surg Endosc.

[37] Ioannou A., Paikousis L., Papastavrou E., Avraamides M.N., Astras G., Charalambous A. (2022). Effectiveness of virtual reality Vs guided imagery on mood changes in cancer patients receiving chemotherapy treatment: a crossover trial. Eur J Oncol Nurs.

[38] Gao J., Liu S., Zhang S. (2022). Pilot study of a virtual reality educational intervention for radiotherapy patients prior to initiating treatment. J Cancer Educ.

[39] Zhou Z., Li J., Wang H. (2023). Experience of using a virtual reality rehabilitation management platform for breast cancer patients: a qualitative study. Support Care Cancer.

[40] Mao W., Chen W., Wang Y. (2024). Effect of virtual reality-based mindfulness training model on anxiety, depression, and cancer-related fatigue in ovarian cancer patients during chemotherapy. Technol Health Care.

[41] Baños R.M., Espinoza M., García-Palacios A. (2013). A positive psychological intervention using virtual reality for patients with advanced cancer in a hospital setting: a pilot study to assess feasibility. Support Care Cancer.

[42] Fahiminia S., Salahiyan A., Norouzi M. (2019). The effectiveness of mindfulness therapy by VR (Virtual-Reality) with a focus on death anxiety in a patient with cerebellar cancer (Case Report). Int Clin Neurosci J.

[43] King A.L., Roche K.N., Leeper H.E. (2023). Feasibility of a virtual reality intervention targeting distress and anxiety symptoms in patients with primary brain tumors: interim analysis of a phase 2 clinical trial. J Neuro-Oncol.

[44] da Silva Alves R., Iunes D.H., Pereira I.C. (2017). Influence of exergaming on the perception of cancer-related fatigue. Games Health J.

[45] Villumsen B.R., Jorgensen M.G., Frystyk J., Hørdam B., Borre M. (2019). Home-based ‘exergaming’ was safe and significantly improved 6-min walking distance in patients with prostate cancer: a single-blinded randomised controlled trial. BJU Int.

[46] Garrett B.M., Tao G., Taverner T., Cordingley E., Sun C. (2020). Patients perceptions of virtual reality therapy in the management of chronic cancer pain. Heliyon.

[47] Basha M.A., Aboelnour N.H., Alsharidah A.S., Kamel F.H. (2022). Effect of exercise mode on physical function and quality of life in breast cancer–related lymphedema: a randomized trial. Support Care Cancer.

[48] Scates D., Dickinson J.I., Sullivan K., Cline H., Balaraman R. (2020). Using nature-inspired virtual reality as a distraction to reduce stress and pain among cancer patients. Environ Behav.

[49] Schneider S.M., Prince-Paul M., Allen M.J., Silverman P., Talaba D. (2004). Virtual reality as a distraction intervention for women receiving chemotherapy. Oncol Nurs Forum.

[50] Schneider S.M., Hood L.E. (2007).

[51] Birkhoff S.D. (2021). The effects of virtual reality on anxiety and self-efficacy among patients with cancer: a pilot study. Oncol Nurs Forum.

[52] Torres García A., Morcillo Serra C., Argilés Huguet M., González Gardó L., Abad Esteve A., Ramos Quiroga J.A. (2023). Efficacy of a virtual reality intervention for reducing anxiety, depression, and increasing disease coping in patients with breast cancer before their first chemotherapy dose. Cogn Ther Res.

[53] Burrai F., Ortu S., Marinucci M., De Marinis M.G., Piredda M. (2023). Effectiveness of immersive virtual reality in people with cancer undergoing antiblastic therapy: a randomized controlled trial. Sem Oncol Nurs.

[54] Uslu A., Arslan S. (2023). The effect of using virtual reality glasses on anxiety and fatigue in women with breast cancer receiving adjuvant chemotherapy: a pretest-posttest randomized controlled study. Sem Oncol Nurs.

[55] Schneider S.M., Ellis M., Coombs W.T., Shonkwiler E.L., Folsom L.C. (2003). Virtual reality intervention for older women with breast cancer. CyberPsychol Behav.

[56] Glennon C. (2018). Use of virtual reality to distract from pain and anxiety. Oncol Nurs Forum.

[57] Chirico A., D’Aiuto M., Pinto M. (2016). Intelligent Interactive Multimedia Systems and Services.

[58] Reynolds L.M., Cavadino A., Chin S. (2022). The benefits and acceptability of virtual reality interventions for women with metastatic breast cancer in their homes; a pilot randomised trial. BMC cancer.

[59] Jadmiko A.W., Kristina T.N., Sujianto U., Prajoko Y.W., Dwiantoro L., Widodo A.P. (2022). A Quasi-experimental of a virtual reality content intervention for level of comfort of indonesian cancer patients. CIN: Comput Informat Nurs.

[60] Niki K., Okamoto Y., Maeda I. (2019). A novel palliative care approach using virtual reality for improving various symptoms of terminal cancer patients: a preliminary prospective, multicenter study. J Palliat Med.

[61] Kaneda M., Oyama H., Katsumata N. (1999). VR intervention therapy for emotion related cancer chemotherapy side effects. Int Conf Artif Real Telexistence.

[62] Horesh D., Kohavi S., Shilony-Nalaboff L. (2022).

[63] Hoffman A.J., Brintnall R.A., Brown J.K. (2013). Too sick not to exercise: using a 6-week, home-based exercise intervention for cancer-related fatigue self-management for postsurgical non–small cell lung cancer patients. Cancer Nurs.

[64] Janssen A., Fletcher J., Keep M. (2022). Experiences of patients undergoing chemotherapy with virtual reality: mixed methods feasibility study. JMIR Serious Games.

[65] Persson J., Clifford D., Wallergård M., Sandén U. (2021). A virtual smash room for venting frustration or just having fun: participatory design of virtual environments in digitally reinforced cancer rehabilitation. JMIR Rehabil Assist Technol.

[66] Cartujano-Barrera F., Sanderson Cox L., Arana-Chicas E. (2020). Feasibility and acceptability of a culturally-and linguistically-adapted smoking cessation text messaging intervention for Latino smokers. Front Public Health.

[67] Giannelli A., Moscato S., Ostan R. (2024). Virtual reality for advanced cancer patients assisted at home: a randomized controlled interventional study. Psycho-Oncology.

[68] Moscato S., Sichi V., Giannelli A. (2021). Virtual reality in home palliative care: brief report on the effect on cancer-related symptomatology. Front Psychol.

[69] Wong C.L., Li C.K., Choi K.C. (2022). Effects of immersive virtual reality for managing anxiety, nausea and vomiting among paediatric cancer patients receiving their first chemotherapy: an exploratory randomised controlled trial. Eur J Oncol Nurs.

[70] Wilson K., Scorsone G. (2021). The use of virtual reality technologies to reduce anxiety and improve experience in chemotherapy patients during treatment. Front Virtual Real.

[71] Feyzioğlu Ö, Dinçer S., Akan A., Algun Z.C. (2020). Is Xbox 360 Kinect-based virtual reality training as effective as standard physiotherapy in patients undergoing breast cancer surgery?. Support Care Cancer.

[72] House G., Burdea G., Grampurohit N. (2016). A feasibility study to determine the benefits of upper extremity virtual rehabilitation therapy for coping with chronic pain post-cancer surgery. Br J Pain.

[73] Mohammad E.B., Ahmad M. (2019). Virtual reality as a distraction technique for pain and anxiety among patients with breast cancer: a randomized control trial. Palliat Support Care.

[74] Atef D., Elkeblawy M.M., El-Sebaie A., Abouelnaga W.A.I. (2020). A quasi-randomized clinical trial: virtual reality versus proprioceptive neuromuscular facilitation for postmastectomy lymphedema. J Egypt Nat Cancer Institute.

[75] Chuan A., Hatty M., Shelley M. (2023). Feasibility of virtual reality-delivered pain psychology therapy for cancer-related neuropathic pain: a pilot randomised controlled trial. Anaesthesia.

[76] Zhao Q., Liu B., Sun Q., Jin Y. (2023). Development and validation of a cost-effective virtual reality educational tool to reduce anxiety and improve set-up accuracy in radiotherapy patients. Cancer Med.

[77] Park H-Y, Nam K.E., Lim J-Y, Yeo S.M., Lee J.I., Hwang J.H. (2023). Real-time interactive digital health care system for postoperative breast cancer patients: a randomized controlled trial. Telemed e-Health.

[78] Schrempf M.C., Petzold J., Petersen M.A. (2022). A randomised pilot trial of virtual reality-based relaxation for enhancement of perioperative well-being, mood and quality of life. Sci Rep.

[79] Fabi A., Fotia L., Giuseppini F. (2022). The immersive experience of virtual reality during chemotherapy in patients with early breast and ovarian cancers: the patient’s dream study. Front Oncol.

[80] Song R., Chen Q., Zhang Y. (2022). Psychophysiological restorative potential in cancer patients by virtual reality (VR)-based perception of natural environment. Front Psychol.

[81] Zhou Z., Li J., Wang H., Luan Z., Li Y., Peng X. (2021). Upper limb rehabilitation system based on virtual reality for breast cancer patients: development and usability study. PloS One.

[82] Zhu E., Hadadgar A., Masiello I., Zary N. (2014). Augmented reality in healthcare education: an integrative review. PeerJ.

[83] Thomas T.H., Go K., Go K. (2022). Empowerment through technology: a systematic evaluation of the content and quality of mobile applications to empower individuals with cancer. Int J Med Informat.

[84] Benson J., Bhandari P., Lui N. (2022).

[85] Bu X., Ng P.H., Xu W. (2022). The effectiveness of virtual reality–based interventions in rehabilitation management of breast cancer survivors: systematic review and meta-analysis. JMIR Serious Games.

[86] Shingler S.L., Bennett B.M., Cramer J.A., Towse A., Twelves C., Lloyd A.J. (2014). Treatment preference, adherence and outcomes in patients with cancer: literature review and development of a theoretical model. Curr Med Res Opin.

[87] Theofilou P., Panagiotaki H. (2012). A literature review to investigate the link between psychosocial characteristics and treatment adherence in cancer patients. Oncol Rev.

[88] Tolotti A., Barello S., Vignaduzzo C. (2022). Patient engagement in oncology practice: a qualitative study on patients' and nurses' perspectives. Int J Environ Res Public Health.

[89] Kenner B.J., Abrams N.D., Chari S.T. (2021). Early detection of pancreatic cancer: applying artificial intelligence to electronic health records. Pancreas.

[90] Kemeny A, George P, Colombet F, Merienne F, New VR navigation techniques to reduce cybersickness, 2017. https://hal.science/hal-01779593/document. Accessed November 18, 2025.

[91] Budhiraja P, Miller MR, Modi AK, Forsyth D. Rotation blurring: use of artificial blurring to reduce cybersickness in virtual reality first person shooters. *arXiv preprint* arXiv:1710.02599. 2017.

[92] Groth C., Tauscher J-P, Heesen N., Castillo S., Magnor M. (2021).

