# Supplementary material for: Extended reality in supporting cancer patients and survivors: A systematic review on the benefits and challenges across the cancer care continuum
Source: Future Healthc J. 2025 Oct 28;13(1):100483. doi: 10.1016/j.fhj.2025.100483 (PMC12769808; doi:10.1016/j.fhj.2025.100483)
Supplement: Supplementary file 1 [file mmc1.docx]

**Appendix 1. Selection criteria and data extraction process**

- 1. **Selection criteria**
     1. **Inclusion criteria**

In this review, we considered studies that (1) were empirical (quantitative or qualitative), (2) reported outcomes related to adult cancer patients (18 years+), (3) involved the use of one of the extended reality technologies, (4) were peer-reviewed, and (5) written in English.

- - 1. **Exclusion criteria**

We excluded any articles that (1) focused solely on cancer care providers or pediatric patients, (2) did not involve the use of extended reality tools, (3) did not report on intervention outcomes, (4) were written in other languages than English, and (5) were not empirical (e.g., opinion papers, editorials, commentaries, and reviews). To ensure methodological rigor, we restricted our search to peer-reviewed empirical studies and did not include grey literature such as preprints, conference abstracts, dissertations, or non-peer-reviewed reports. This decision was made to enhance study quality and reproducibility by ensuring that only rigorously vetted research was included.

- 1. **Selection and screening process**

The initial search yielded 34805 records (see Figure 2). After removing duplicates (n=9026), we screened the titles and abstracts for eligibility. Of these articles, n=321 were written in a language other than English and were excluded. After the title and abstract screening, 191 articles were downloaded for a full-text review, and sixty-two were determined to be eligible for review. The selection was made separately by two authors, and at each step of the process, discrepancies were discussed until a consensus was reached. All screening was conducted in Microsoft Excel: each reviewer independently screened titles/abstracts and full texts in separate worksheets. To enhance objectivity, the reviewers performed initial screening blinded to each other’s decisions; results were then compared, and any discrepancies were resolved through discussion.

**Figure 2.** PRISMA flowchart for the search process

- 1. **Data Extraction and Synthesis**

A data extraction form was prepared to help guide the synthesis process. The data extraction included information related to the intervention (study design, sample size, randomized control trial or not), the population (demographics if reported, type of cancer, stage of cancer), the type of XR tool used (type of technology, brand, barriers of success and side effects, functions, effectiveness compared to traditional therapies if reported), and the health outcomes (health outcomes measured). The review team discussed and synthesized information in an iterative process. We also reported the stage of the support given following the Cancer Care Continuum framework, categorizing the interventions based on prevention, early detection and diagnosis, treatment, palliative care, and survivorship (Chan et al., 2023). To ensure good quality of reporting, all the selection steps were subject to consensus by all the authors.

- 1. **Quality assessment and risk of bias**

We also assessed the study’s risk of bias using the Mixed Methods Appraisal Tool (MMAT) (REF). The MMAT assists in determining the quality of qualitative, quantitative, and mixed-method studies by evaluating specific methodological criteria appropriate for the study design. If a study fully met all evaluation criteria, the likelihood of bias was low, and the quality grade was "A." If these criteria were partially met, the probability of bias was determined to be moderate, and the quality grade was "B." If these criteria were not met, the probability of bias was high, and the quality grade was "C". Articles with an overall quality rating of A or B were included in the final review. All articles reviewed were rated as level A or B and were included in the review. Appendix 2 summarizes the results of the methodological appraisal.

**Appendix 2. MMAT Risk appraisal results**

|  | **SCREENING QUESTIONS** | | **1. QUALITATIVE STUDIES** | | | | | **2. RANDOMIZED CONTROLLED TRIALS** | | | | | **3. NON-RANDOMIZED STUDIES** | | | | | **5. MIXED METHODS STUDIES** | | | | | **MMAT Score** | **Overall quality** |
| --- | --- | --- | --- | --- | --- | --- | --- | --- | --- | --- | --- | --- | --- | --- | --- | --- | --- | --- | --- | --- | --- | --- | --- | --- |
| Title | S1 | S2 | 1.1 | 1.2 | 1.3 | 1.4 | 1.5 | 2.1 | 2.2 | 2.3 | 2.4 | 2.5 | 3.1 | 3.2 | 3.3 | 3.4 | 3.5 | 5.1 | 5.2 | 5.3 | 5.4 | 5.5 |  |  |
| (Oyama et al., 1999) | Y | Y |  |  |  |  |  |  |  |  |  |  | Y | Y | Y | CT | Y |  |  |  |  |  | 6 | very high |
| (Kaneda et al., 1999) | Y | Y |  |  |  |  |  |  |  |  |  |  | Y | Y | Y | CT | Y |  |  |  |  |  | 5 | very high |
| (Oyama et al., 2000) | Y | Y |  |  |  |  |  | Y | Y | Y | CT | Y |  |  |  |  |  |  |  |  |  |  | 6 | very high |
| (Schneider et al., 2003) | Y | Y |  |  |  |  |  |  |  |  |  |  | Y | Y | Y | CT | Y |  |  |  |  |  | 6 | very high |
| (Schneider et al., 2004) | Y | Y |  |  |  |  |  |  |  |  |  |  | Y | Y | Y | CT | Y |  |  |  |  |  | 6 | very high |
| (Schneider & Hood, 2007) | Y | Y |  |  |  |  |  | CT | Y | Y | N | Y |  |  |  |  |  |  |  |  |  |  | 5 | very high |
| (Schneider et al., 2011) | Y | Y |  |  |  |  |  | Y | Y | Y | N | Y |  |  |  |  |  |  |  |  |  |  | 6 | very high |
| (Baños et al., 2013) | Y | Y |  |  |  |  |  |  |  |  |  |  |  |  |  |  |  | Y | Y | Y | N | CT | 5 | very high |
| (Hoffman et al., 2013) | Y | Y |  |  |  |  |  |  |  |  |  |  | N | Y | Y | N | Y |  |  |  |  |  | 5 | very high |
| (Hoffman et al., 2014) | Y | Y |  |  |  |  |  |  |  |  |  |  | N | Y | Y | N | Y |  |  |  |  |  | 5 | very high |
| (Tsuda et al., 2016) | Y | Y |  |  |  |  |  |  |  |  |  |  | Y | Y | Y | N | Y |  |  |  |  |  | 5 | very high |
| (Chirico et al., 2016) | Y | Y |  |  |  |  |  | CT | Y | Y | N | Y |  |  |  |  |  |  |  |  |  |  | 5 | very high |
| (House et al., 2016) | Y | Y |  |  |  |  |  |  |  |  |  |  | N | Y | Y | N | Y |  |  |  |  |  | 5 | very high |
| (da Silva Alves et al., 2017) | Y | Y |  |  |  |  |  |  |  |  |  |  | Y | Y | Y | N | Y |  |  |  |  |  | 6 | very high |
| (da Silva Alves et al., 2018) | Y | Y |  |  |  |  |  |  |  |  |  |  | Y | Y | Y | N | Y |  |  |  |  |  | 6 | very high |
| (Glennon, 2018) | Y | Y |  |  |  |  |  |  |  |  |  |  | Y | Y | Y | N | Y |  |  |  |  |  | 6 | very high |
| (Jimenez et al., 2018) | Y | Y |  |  |  |  |  |  |  |  |  |  | Y | Y | Y | N | Y |  |  |  |  |  | 6 | very high |
| (Mohammad & Ahmad, 2019) | Y | Y |  |  |  |  |  | Y | Y | Y | N | Y |  |  |  |  |  |  |  |  |  |  | 6 | very high |
| (Fahiminia et al., 2019) | Y | Y |  |  |  |  |  |  |  |  |  |  | no | yes | yes | no | no |  |  |  |  |  | 4 | high |
| (Cîmpean, 2019) | Y | Y |  |  |  |  |  |  |  |  |  |  | yes | yes | yes | no | yes |  |  |  |  |  | 6 | very high |
| (Niki et al., 2019) | Y | Y |  |  |  |  |  |  |  |  |  |  | yes | yes | yes | yes | yes |  |  |  |  |  | 7 | very high |
| (Villumsen et al., 2019) | Y | Y |  |  |  |  |  | yes | yes | yes | no | yes |  |  |  |  |  |  |  |  |  |  | 6 | very high |
| (Garrett et al., 2020) | Y | Y |  |  |  |  |  |  |  |  |  |  |  |  |  |  |  | Y | Y | Y | N | Y | 6 | very high |
| (Atef et al., 2020) | Y | Y |  |  |  |  |  |  |  |  |  |  | Y | Y | Y | N | Y |  |  |  |  |  | 6 | very high |
| (Chirico et al., 2020) | Y | Y |  |  |  |  |  | CT | Y | Y | N | Y |  |  |  |  |  |  |  |  |  |  | 5 | very high |
| (Feyzioğlu et al., 2020) | Y | Y |  |  |  |  |  | Y | Y | Y | Y | Y |  |  |  |  |  |  |  |  |  |  | 7 | very high |
| (Scates et al., 2020) | Y | Y |  |  |  |  |  |  |  |  |  |  | Y | Y | Y | N | Y |  |  |  |  |  | 6 | very high |
| (Turrado et al., 2021) | Y | Y |  |  |  |  |  | Y | Y | Y | N | Y |  |  |  |  |  |  |  |  |  |  | 6 | very high |
| (Ashley Verzwyvelt et al., 2021) | Y | Y |  |  |  |  |  |  |  |  |  |  | Y | Y | Y | N | Y |  |  |  |  |  | 6 | very high |
| (Persson et al., 2021) | Y | Y |  |  |  |  |  |  |  |  |  |  | Y | Y | Y | N | Y |  |  |  |  |  | 6 | very high |
| (Basha et al., 2022) | Y | Y |  |  |  |  |  | Y | Y | Y | Y | Y |  |  |  |  |  |  |  |  |  |  | 7 | very high |
| (Turrado et al., 2021) | Y | Y |  |  |  |  |  | Y | Y | Y | N | Y |  |  |  |  |  |  |  |  |  |  | 6 | very high |
| (Zhou et al., 2021) | Y | Y |  |  |  |  |  |  |  |  |  |  | Y | Y | Y | N | Y |  |  |  |  |  | 6 | very high |
| (Kelleher et al., 2022) | Y | Y |  |  |  |  |  |  |  |  |  |  |  |  |  |  |  | Y | Y | Y | N | Y | 6 | very high |
| (Horesh et al., 2022) | Y | Y |  |  |  |  |  |  |  |  |  |  | Y | Y | Y | N | Y |  |  |  |  |  | 6 | very high |
| (Horesh et al., 2022) | Y | Y |  |  |  |  |  | Y | Y | Y | Y | Y |  |  |  |  |  |  |  |  |  |  | 7 | very high |
| (Song et al., 2022) | Y | Y |  |  |  |  |  | Y | Y | Y | Y | Y |  |  |  |  |  |  |  |  |  |  | 7 | very high |
| (Austin et al., 2022) | Y | Y |  |  |  |  |  | Y | Y | Y | N | Y |  |  |  |  |  |  |  |  |  |  | 6 | very high |
| (Schrempf et al., 2022) | Yes | Y |  |  |  |  |  | Y | Y | Y | Y | Y |  |  |  |  |  |  |  |  |  |  | 7 | very high |
| (Jadmiko et al., 2022) | Y | Y |  |  |  |  |  |  |  |  |  |  | Y | Y | Y | N | Y |  |  |  |  |  | 6 | very high |
| (Ioannou et al., 2022) | Y | Y |  |  |  |  |  | Y | Y | Y | N | Y |  |  |  |  |  |  |  |  |  |  | 6 | very high |
| Methods Feasibility Study | Y | Y |  |  |  |  |  |  |  |  |  |  |  |  |  |  |  | Y | Y | Y | N | N | 5 | very high |
| (Janssen et al., 2022) | Y | Y |  |  |  |  |  | Y | Y | Y | N | Y |  |  |  |  |  |  |  |  |  |  | 6 | very high |
| (O'Gara et al., 2022) | Y | Y |  |  |  |  |  |  |  |  |  |  |  |  |  |  |  | Y | Y | Y | Y | Y | 7 | very high |
| (Wong et al., 2022) | Y | Y |  |  |  |  |  |  |  |  |  |  |  |  |  |  |  | Y | Y | Y | N | Y | 6 | very high |
| (X. Zhang et al., 2022) | Y | Y |  |  |  |  |  | Y | Y | Y | N | Y |  |  |  |  |  |  |  |  |  |  | 6 | very high |
| (Park et al., 2023) | Y | Y |  |  |  |  |  | Y | Y | Y | Y | Y |  |  |  |  |  |  |  |  |  |  | 7 | very high |
| (Chuan et al., 2023) | Y | Y |  |  |  |  |  | Y | Y | Y | Y | Y |  |  |  |  |  |  |  |  |  |  | 7 | very high |
| (King et al., 2023) | Y | Y |  |  |  |  |  |  |  |  |  |  |  |  |  |  |  | Y | Y | Y | N | Y | 6 | very high |
| (Torres García et al., 2024) | Y | Y |  |  |  |  |  | Y | Y | Y | N | Y |  |  |  |  |  |  |  |  |  |  | 6 | very high |
| (Burrai et al., 2023) | Y | Y |  |  |  |  |  | Y | Y | Y | N | Y |  |  |  |  |  |  |  |  |  |  | 6 | very high |
| (Mao et al., 2024) | Y | Y |  |  |  |  |  |  |  |  |  |  | Y | Y | Y | N | Y |  |  |  |  |  | 6 | very high |
| (Zhao et al., 2023) | Y | Y |  |  |  |  |  | Y | Y | Y | N | Y |  |  |  |  |  |  |  |  |  |  | 6 | very high |
| (Uslu & Arslan, 2023) | Y | Y |  |  |  |  |  | Y | Y | Y | N | Y |  |  |  |  |  |  |  |  |  |  | 6 | very high |
| (Uslu & Arslan, 2023) | Y | Y |  |  |  |  |  | Y | Y | Y | N | Y |  |  |  |  |  |  |  |  |  |  | 6 | very high |
| (Zhou et al., 2023) | Y | Y | Y | Y | Y | Y | Y |  |  |  |  |  |  |  |  |  |  |  |  |  |  |  | 7 | very high |
| (Moscato et al., 2021) | Y | Y |  |  |  |  |  |  |  |  |  |  | Y | Y | Y | N | Y |  |  |  |  |  | 6 | very high |
| (Wilson & Scorsone, 2021) | Y | Y |  |  |  |  |  |  |  |  |  |  | Y | Y | Y | N | Y |  |  |  |  |  | 6 | very high |
| (Fabi et al., 2022) | Y | Y |  |  |  |  |  | CT | Y | Y | N | Y |  |  |  |  |  |  |  |  |  |  | 5 | very high |
| (Giannelli et al., 2024) | Y | Y |  |  |  |  |  | Y | Y | Y | Y | Y |  |  |  |  |  |  |  |  |  |  | 7 | very high |
| S1. Are there clear research questions?  S2. Do the collected data allow us to address the research questions?  1.1. Is the qualitative approach appropriate to answer the research question?  1.2. Are the qualitative data collection methods adequate to address the research question?  1.3. Are the findings adequately derived from the data?  1.4. Is the interpretation of results sufficiently substantiated by data?  1.5. Is there coherence between qualitative data sources, collection, analysis and interpretation?  2.1. Is randomization appropriately performed?  2.2. Are the groups comparable at baseline?  2.3. Are there complete outcome data?  2.4. Are outcome assessors blinded to the intervention provided?  2.5 Did the participants adhere to the assigned intervention?  3.1. Are the participants representative of the target population?  3.2. Are measurements appropriate regarding both the outcome and intervention (or exposure)?  3.3. Are there complete outcome data?  3.4. Are the confounders accounted for in the design and analysis?  3.5. During the study period, is the intervention administered (or exposure occurred) as intended?  5.1. Is there an adequate rationale for using a mixed methods design to address the research question?  5.2. Are the different components of the study effectively integrated to answer the research question?  5.3. Are the outputs of the integration of qualitative and quantitative components adequately interpreted?  5.4. Are divergences and inconsistencies between quantitative and qualitative results adequately addressed?  5.5. Do the different components of the study adhere to the quality criteria of each tradition of the methods involved?  CT: CAN’T TELL  Y: Yes  N: No | | | | | | | | | | | | | | | | | | | | | | | | |

**Appendix 3. Study and participants' information**

**Descriptive results**

We included 62 studies in the review. The included distribution over time shows an increasing interest in immersive technology (Figure 3). For instance, while only 3 studies used extended reality to support cancer patients before 2000, this number increased substantially to 59 studies between 2000 and 2024. Among the included studies, 27 used a randomized controlled trial design. The sample size across these 27 trials ranged between 1 and 196 participants (mean=50.44). The XR interventions reviewed included a variety of cancer patients. Nineteen studies presented technologies that were designed for more than two types of cancer. Most studies focused on breast cancer patients (N =24). Five of the studies included patients with ovarian cancer, four focused on colorectal cancer, two concentrated on patients with lung cancer, and two included patients with hematologic cancers.

**Technology tools and equipment used**

Of the 62 studies, 2 used augmented reality technology (AR) tools and 60 used virtual reality (VR) experiments. Most of them used headsets such as Mirage Solo, Oculus Go/Quest/Rift, Samsung Gear, Pico Neo4, HTC Vive, i-Glasses, and Google Cardboard, among others). While 38 technologies were mainly designed for clinic use, 20 were for home use, and 5 were created for both. More details on the XR interventions are summarized in Appendix 4.

**Technology functions of support to cancer patients across the cancer care continuum.**

Of the studies reviewed, most XR interventions were used to support cancer patients during the active treatment (n=40/62), palliative, and survivorship phases (n=21/62) of care. Only one study focused on supporting patients at diagnosis, and none provided patient support approaches for prevention or early detection. Figure 4 summarizes the most common patient care endpoints targeted by XR interventions.

|  | **Objective of the paper** | **Study design** | **Is it a randomized control trial? yes or no** | **Saple** | **What is the population age range or mean?** | **What is the type of cancer of the population of the study?** | **What is their stage of cancer, if available?** |
| --- | --- | --- | --- | --- | --- | --- | --- |
| (Schneider et al., 2003) | examine the effects of a virtual reality distraction intervention on chemotherapy-related symptom distress levels in older women with breast cancer. | a cross-over design. | no | 16 | The population age range is 50–77 years. | Breast cancer | Not mentioned |
| (Moscato et al., 2021) | assess the effect of an immersive VR-based intervention conducted at home on anxiety, depression, and pain over 4 days and to evaluate the short-term effect of VR sessions on cancer-related symptomatology in advanced cancer patients. | a pre-post single-arm study. | no | 14 | The population age range in the study is from 18 to 70 years. The mean age of participants is 47.2 ± 14.2 years. | Cancer all types | advanced |
| (Kelleher et al., 2022) | examine the feasibility, acceptability, safety, and impact of a 30-minute virtual reality session using a virtual underwater/sea environment (VR Blue) for reducing pain and pain-related symptoms in patients with advanced-stage colorectal cancer. | a pilot trial. | no | 20 | The population age range in the study is 18-85 years. | Colorectal cancer | Stage IV |
| (Horesh et al., 2022) | assess the effectiveness of Bubble, a novel artificial intelligence–virtual reality (AI–VR) intervention for the treatment of hot flashes in female breast or ovarian cancer patients. | a pilot study. | no | 42 | The population age range is 25–60 years, with a mean age of 47 years. | Breast and ovarian cancer | ranged from 1 to 4. |
| (Wilson & Scorsone, 2021) | investigate the impact of nature scenes in a 3D virtual reality environment on patients receiving chemotherapy treatment. | a pre and post-intervention assessment | no | 22 | The population age range is between 32 and 78 years of age. | breast cancer | Not mentioned |
| (Fabi et al., 2022) | evaluate the impact of Virtual Reality Experience (VRE) as a distraction therapy on psychological distress, anxiety, and quality of life in patients affected by breast or ovarian cancer during their first cycle of adjuvant chemotherapy. | a two-arm randomized controlled trial. | yes | 44 | The population age range is 37-71 years. | breast and ovarian cancers | Stage I–III |
| (Fahiminia et al., 2019) | explore the efficiency of mindfulness therapy with the aid of virtual reality (VR), focusing on minimizing anxiety, especially death anxiety, in a patient with cerebellar cancer. | a single case study. | no | 1 | 50-year-old | brain cancer | Not mentioned |
| (Reynolds et al., 2022) | assess whether virtual reality (VR) should be pursued as a feasible and acceptable adjunctive therapy to alleviate physical and psychological symptoms in women with metastatic breast cancer (MBC). | a pilot randomized trial. | yes | 38 | Not specified. | metastatic breast cancer | metastatic breast cancer (MBC) |
| (Park et al., 2023) | investigate the effect of hospital-home-linked rehabilitation therapy using an AR-based digital health care system (UINCARE Home+) in postoperative patients with breast cancer. | a prospective, multicenter, assessor-blinded, randomized controlled trial. | yes | 100 | 20–70 years | Breast cancer | Not mentioned |
| (Song et al., 2022) | explore the psychophysiological recovery of cancer patients through the perception of different types of natural environments using virtual reality (VR), and to determine the optimal frequency of interventions needed to achieve maximum restoration. | experimental design. | yes | 63 | The population age range in the study is from 59.48 ± 12.06 years to 61.31 ± 15.10 years. | esophageal or gastrointestinal cancer | Not mentioned |
| (Garrett et al., 2020) | explore the experiences of individuals with chronic pain associated with a cancer diagnosis in using a daily VR-based self-administered home therapy for one month as an adjunctive measure to help manage pain, determine their perceptions of the value of such VR therapy, and evaluate the effects of VR interventions based upon either cognitive problem-solving or mindfulness and relaxation in providing relief from pain as well as the aspects that supported or detracted from pain management. | a qualitative study using focus groups. | yes | 12 | The population age range is 37-73 years. | Cancer, many types (abdominal, breast, bone, colon, kidney, lung, non-Hodgkin's lymphoma, and throat cancers) | Not mentioned |
| (Chuan et al., 2023) | evaluate the feasibility, acceptability, recruitment rates, and risk of cybersickness of a bespoke virtual reality-delivered pain therapy software program designed to help cancer patients manage neuropathic pain. Secondary aims include assessing clinical outcomes such as opioid consumption, pain severity, pain interference, and global quality of life scores. | a single-center, prospective, pilot randomized controlled trial. | yes | 39 | The population age range is ≥ 18 years. | Cancer all types | Not mentioned |
| (King et al., 2023) | explore the feasibility and acceptability of a virtual reality relaxation intervention for primary brain tumor patients at the time of clinical evaluation. | a interim analysis of a phase 2 clinical trial. | no | 20 | 65% of participants were ≤ 50 years. | Primary brain tumor | Not mentioned |
| (Cartujano-Barrera et al., 2020) | determine the feasibility and preliminary effectiveness of larger randomized controlled trials of 3D head-mounted virtual reality (VR) for managing cancer pain in adults receiving palliative care. | a single-session randomized cross-over trial. | no | 13 | Not specified. | Cancer all types | advanced and terminal stages of cancer. |
| (Turrado et al., 2021) | determine the presence of pre- and post-exposure anxiety in patients undergoing surgery for colorectal cancer using virtual reality, measured by the State-Trait Anxiety Inventory Scale (STAI-S) and Hospital Anxiety and Depression Scale (HADS). | a single-center prospective randomized clinical trial. | yes | 126 | Not specified. | Colorectal cancer | Not mentioned |
| (Torres García et al., 2023) | compare the level of anticipative anxiety and depression in patients prepared for their first dose of chemotherapy for breast cancer with virtual reality versus a standard psychoeducational intervention. | single-center, comparative, and randomized. | yes | 133 | The population age range is 30–82 years, with a mean age of 49. | Breast cancer | Not mentioned |
| (Ashley Verzwyvelt et al., 2021) | determine if a biophilic Green Therapy or Virtual Reality environment can decrease an oncology patient’s pain and distress while receiving chemotherapy. | a case-crossover pilot study. | no | 33 | Not specified. | Cancer, different types | Not mentioned |
| (Burrai et al., 2023) | assess the effects of immersive Virtual Reality in people with cancer undergoing antiblastic therapy, on anxiety, fatigue, and pain. | a randomized controlled three-arm trial. | yes | 74 | Not specified. | Cancer all types | Not mentioned |
| (Mao et al., 2023) | explore the impact of a training mode based on the integration of virtual reality technology and mindfulness on anxiety, depression, and cancer-related fatigue in ovarian cancer patients during chemotherapy. | a self-controlled design. | no | 48 | The population age range is 18–69 years. | ovarian cancer | Stage II-IV |
| (Schneider et al., 2011) | explore the influence of age, gender, state anxiety, fatigue, and cancer diagnosis on time perception in cancer patients receiving intravenous chemotherapy with a VR distraction intervention within the framework of the PA cognitive model of time perception and predict the effects of these variables on the difference between the actual time elapsed. In contrast, patients received chemotherapy while immersed in a VR environment and their retrospective estimates of elapsed time. | a secondary analysis of pooled data from three trials with a crossover design | yes | 137 | The population age range is 27–78 years. | breast, lung, or colon cancer | Not mentioned |
| (Zhao et al., 2023) | develop and validate a cost-effective virtual reality educational tool to reduce anxiety and improve set-up accuracy for radiotherapy patients. | a prospective pilot study. | Yes | 120 | The population age range is >18 years. | thoracic or abdominal cancer | Ranged from 1 to 4 |
| (Persson et al., 2021) | understand attitudes to, and some effects of, using a virtual smash room application in cancer rehabilitation, as well as eliciting ideas for other virtual environments that patients would desire. | a participatory design. | no | 101 | Not specified. | Cancer all types | Not mentioned |
| (Schrempf et al., 2022) | investigate the feasibility and clinical effects of a VR intervention on quality of life, well-being, and mood in cancer patients undergoing surgery compared to a non-VR intervention and a control group. | a randomized pilot trial. | yes | 54 | Not specified. | Colorectal cancer | Advanced cancer |
| (Jadmiko et al., 2022) | determine the effect of virtual reality content on the comfort level of cancer patients. | a quasi-experimental non-equivalent control group design. | no | 60 | 18-60 years | rectal cancer | Stages II-IV |
| (Baños et al., 2013) | explore the feasibility of a psychological intervention using virtual reality to induce positive emotions in adult hospitalized patients with metastatic cancer, and to assess the patient's satisfaction and perceived utility of the intervention. | a single-group descriptive pilot study. | no | 19 | The population age range in the study is from 29 to 85 years old. | Cancer all types | advanced stages of cancer. |
| (Cîmpean, 2019) | compare the effectiveness of Cognitive Behavioral Therapy with Virtual Reality (CBT VR) with standard Cognitive Behavioral Therapy (CBT) in reducing acute cancer-related anxiety and depressive symptoms in patients who have undergone cervical cancer surgery. | a non-randomized pilot study with two groups | no | 20 | Not specified. | cervical cancer | Not mentioned |
| (Niki et al., 2019) | verify whether simulated travel using virtual reality (VR travel) is efficacious in improving symptoms in terminal cancer patients. | a prospective, multicenter, single-arm study. | no | 20 | The average age of the participants was 72.3 years. | Cancer different types | Terminal stage. |
| (Tsuda et al., 2016) | investigate the feasibility and safety of virtual reality exercise intervention using Nintendo Wii Fit in elderly patients with hematologic malignancies receiving chemotherapy. | a prospective, single-arm feasibility study. | no | 16 | 60-76 years | hematologic malignancies | Not mentioned |
| (Uslu & Arslan, 2023) | determine the effect of using virtual reality glasses on anxiety and fatigue in women with breast cancer receiving adjuvant chemotherapy. | a pretest-posttest randomized controlled study. | yes | 66 | Not specified. | Breast cancer | Not mentioned |
| (Oyama et al., 1999) | evaluate the psycho-oncological effectiveness of the Bedside Wellness System, a virtual forest walk system designed to improve the quality of life of bedridden cancer patients by incorporating virtual reality technology to support their mental health. | a one-arm pilot study. | no | 22 | The population age range is 33-75 years. | breast and ovarian cancer. | Performance states 0-2 are under study. |
| (Kaneda et al., 1999) | determine if virtual reality intervention therapy can reduce anticipatory nausea and vomiting in cancer patients undergoing chemotherapy. | Phase II study | no | 10 | The population age range is between 16 and 70 years. | Hodgkin’s disease and breast cancer | Not mentioned |
| (Oyama et al., 2000) | develop a new treatment using Virtual Reality (VR) technology as an intervention therapy to decrease chemotherapy-related side effects in cancer patients. | a randomized controlled trial. | yes | 30 | The patients were between 29 and 73, averaging 53.5 years. | Cancer different types | Ranged from 1 to 4 |
| (Schneider et al., 2004) | explore the use of virtual reality as a distraction intervention to relieve symptom distress in women receiving chemotherapy for breast cancer. | Crossover study. | no | 20 | 18–55 years of age. | Breast cancer | Not mentioned |
| (Schneider & Hood, 2007) | explore virtual reality (VR) as a distraction intervention to relieve symptom distress in adults receiving chemotherapy treatments for breast, colon, and lung cancer. | Control study | yes | 123 | The average participant was 54 years old. | Cancer different types | Not mentioned |
| (Mao et al., 2024) | explore the impact of a training mode based on the integration of virtual reality technology and mindfulness on anxiety, depression, and cancer-related fatigue in ovarian cancer patients during chemotherapy. | a self-controlled design. | no | 48 | The age range of the population in the study is 18–69 years. | ovarian cancer | Stage II-IV |
| (Hoffman et al., 2014) | Investigate the feasibility and acceptability of a home based rehabilitation exercise intervention for lung cancer patients | A single-arm design | No | 7 | mean: 65 | Lung cancer | 1 to 3 |
| (House et al., 2016) | explore the feasibility of BrightArm Duo therapy for coping with post-surgical chronic pain and associated disability in breast cancer survivors with depression. | Pilot study | No | 6 | 57.8 years (ranging from 22 to 78 years) | breast cancer | Not specified |
| (Glennon, 2018) | determine the effects of a virtual reality intervention on pain and anxiety in patients undergoing a bone marrow aspiration and biopsy procedure. | Quasiexperimental research design | No | 97 | ages ranged from 19–70 years, with a mean age of 50.2 years | hematologic cancers | Not specified |
| (Mohammad & Ahmad, 2019) | assess the effectiveness of immersive virtual reality (VR) distraction technology in reducing pain and anxiety among female patients with breast cancer. | Randomized control trial | Yes | 80 | mean: 51.99 years, with a range from 30 to 70 years | breast cancer | 1 to 4 |
| (Feyzioğlu et al., 2020) | investigate the potential effects of early postoperative virtual reality (VR) therapy on pain, range of motion (ROM), muscle strength, functionality, and fear of movement among breast cancer patients post surgery | Prospective randomized controlled study | Yes | 40 | 30 to 60 years | breast cancer | Not specified |
| (Jimenez et al., 2018) | investigate the impact of a newly developed education tool using the VERT system on breast cancer patients' RT knowledge and anxiety | quasi-experimental design | No | 37 | 35 to 74 years | breast cancer | Not specified |
| (Atef et al., 2020) | compare the therapeutic efficiency of virtual reality (VR) and proprioceptive neuromuscular facilitation (PNF) in treating lymphedema and improving function in unilateral postmastectomy lymphedema (UPML) patients with breast cancer. | quasi-randomized comparative study | No | 30 | mean 54.07 range (40-65) | breast cancer | Not specified |
| (Scates et al., 2020) | determine whether a nature-inspired VR simulation reduced stress and pain levels among patients in a cancer treatment center. | repeated measures experimental design | No | 50 | older than 18 | Not specified | Not specified |
| (Basha et al., 2022) | Compare the effects of virtual reality (VR) training and resistance exercises training on lymphedema symptom severity as well as physical functioning and QoL in women with breast cancer–related lymphedema (BCRL). | Single blinded randomized trial | Yes | 60 | Older than 30 | breast cancer | Not specified |
| (Gao et al., 2022) | investigate the impact of a VR radiotherapy (RT) educational system on patients’ understanding and anxiety prior to commencing RT. | single-blinded, randomized controlled trial. | Yes | 60 | 50-59 years | Different types of cancer (lung, breast, esophageal, other) | Not specified |
| (Turrado et al., 2021) | evaluate the effectiveness of exposure to the entire perioperative environment through virtual reality in decreasing the preoperative anxiety of patients with colorectal cancer compared to the standard care. | Single-center randomized clinical trial | Yes | 126 | mean 65 (26-94) | Colorectal cancer | 1 to 3 |
| (Villumsen et al., 2019) | explore the effects of 12 weeks of unsupervised home-based ‘exergaming’ (i.e., technology-driven exercise) compared to usual care on physical function, body composition, quality of life (QoL), and fatigue in patients with prostate cancer on androgen-deprivation therapy (ADT). | assessor-blinded randomised controlled trial | Yes | 46 | mean 67.6 years | Prostate cancer | 1 to 3 |
| (Zhang et al., 2022) | evaluate the effectiveness and feasibility of Managing Cancer and Living Meaningfully based on VR (VR-CALM), which is used to manage expected symptoms of cancer itself, relieve psychological distress, and improve quality of life (QOL) in the Chinese breast cancer survivors (BCs). | non-blind, parallel assignment RCT | Yes | 98 | mean 52.29 years (18-70) | breast cancer | 1 to 4 |
| (Niki et al., 2019) | verify whether simulated travel using virtual reality (VR travel) is efficacious in improving symptoms in terminal cancer patients. | prospective, multicenter, single-arm study | No | 20 | mean 72.3 years (20 to 87) | Different types of cancer | Terminal (4) |
| (Chirico et al., 2016) | To explore the effect of immersive VR on psychological well-being and cognitive functioning in breast cancer patients | Randomized control trial | Yes | 47 | Between 18 and 70 | Breast cancer | Not specified |
| (Chirico et al., 2020) | To investigate the efficacy of VR and music therapy as distraction interventions for alleviating symptom perception and distress in cancer patients | Randomized control trial | Yes | 94 | Mean age SD :55.18 (VR group) 55,7 (Music Therapy) 56,2 (Control Group) | Breast cancer | 1 to 3 |
| (Zhou et al., 2021) | To evaluate the usability and feasibility of a VR-based upper limb rehabilitation system for breast cancer patients | Usability study | No | 15 | Mean age : 54.73±7.78. | Breast cancer | 1 to 3 |
| (Ioannou et al., 2022) | To test whether VR can benefit cancer patients from their interaction with an immersive environment, on their mood and their biophysical parameters, compared to those who will experience a Guided Imagery (GI) intervention. | Randomized crossover trial | Yes | 50 | Mean age: 57 years old (SD = 15.5) | Various types (not specified) | 1 to 4 |
| (Janssen et al., 2022) | Explore feasibility and acceptability of using VR for adult patients undergoing chemotherapy | Mixed methodology | No | 18 | N/A | Various cancers | Not specified |
| (da Silva Alves et al., 2018) | To analyze the effect of exergaming therapy on quality of life as well as the functional well-being (FWB) and physical well-being (PWB) of cancer patients | Quasi-experimental, controlled study | No | 45 | 18 to 80 | Cancer | 0 to 3 |
| (da Silva Alves et al., 2017) | To evaluate the influence of an exergaming protocol on cancer-related fatigue, muscle fatigue, and muscle strength in cancer patients | Quasi-experimental control study | No | 45 | 18 to 80 | Various (gastrointestinal tract, breast, abdominal and pelvic, oropharyngeal, others) | 0 to 3 |
| (Hoffman et al., 2013) | To evaluate the feasibility, acceptability, safety, and changes in study endpoints of a home-based exercise intervention to enhance perceived self-efficacy for cancer-related fatigue (CRF) self-management for persons after thoracotomy for NSCLC transitioning from hospital to home. | Single-arm study | No | 7 | 53 to 73 years old (SD = 64.6) | Non-small cell lung cancer (NSCLC) | 1 to 3 |
| (Giannelli et al., 2024) | Assess the effects of VR, compared to tablet controlled intervention, on anxiety, depression, pain, and short-term psychophysical symptoms in advanced cancer patients assisted at home | Randomized controlled interventional study | Yes | 53 | 55.7 Mean age | Various (gastrointestinal, breast, genital tract, etc.) | Advanced |
| (Zhou et al., 2023) | Understand the user usability experience of breast cancer patients about the postoperative rehabilitation management of upper limb function using virtual reality | Qualitative study | No | 20 | 26 and 68 | Breast cancer | 1 to 3 |
| (Birkhoff, 2021) | To examine the impact of a nurse-led intervention on anxiety levels and perceived self-efficacy to cope in patients receiving first-time chemotherapy using a customized prechemotherapy educational virtual reality (VR) video | Single-group, quasiexperimental pilot stud | No | 35 | 61.8 years (range 37-83 years) | Various cancers | 1 to 6 |
| (O'Gara et al., 2022) | To determine the feasibility and acceptability of a VR intervention incorporating relaxation and compassionate mind training in an oncology setting and evaluate its impact on physical/psychological well-being and quality of life | Phase I: experience based codesign approach, Phase II: mixed methods approach | No | 21 | 48.7 years old | Various cancers | different stages |
| (Shin et al., 2023) | To evaluate the anxiety-reducing effects of virtual reality (VR) on patients with breast cancer undergoing adjuvant radiation therapy (RT) | Randomized controlled trial | Yes | 196 | Mean age 47.4 years (VR group), 47.6 years (control group | breast cancer | 0 to 4 |

**Appendix 4. Extended reality-related outcomes**

| **Citation** | **XR tool** | **Equipment** | **name of the tool/application** | **Tool features** | **Function** | **Clinic or home-based** | **Cancer Care Continuum Phase** | **Barriers to XR use** |
| --- | --- | --- | --- | --- | --- | --- | --- | --- |
| (Schneider et al., 2003) | VR | Nintendo Wii Fit Plus | Not mentioned | Intervention is a virtual reality tool designed to support light-intensity exercise rehabilitation for post-thoracotomy NSCLC patients. It provides a home-based incremental walking exercise with initial set-up by nurses. | Anxiety and stress management | **Home** | Treatment | Not specified |
| (Moscato et al., 2021) | VR | BrightArm Duo Rehabilitation System: Developed by Bright Cloud International Corp, NJ. | The BrightArm Duo Rehabilitation System | Engages patients in upper body bimanual exercises to improve strength, range of motion, and endurance. It also incorporates tasks on cognitive training to enhance executive function, memory and attention. | Symptom tracking and management, Education and information, Pain management | **Clinic** | Palliative care and Survivorship | Sickness. Some participants reported pain or discomfort in their upper body |
| (Kelleher et al., 2022) | VR | ezVision X4 virtual reality goggles | Not mentioned | ezVision X4 virtual reality goggles accompanied by soothing music heard through built-in earphones. The immersive experience aimed to distract patients from the procedure by blocking out negative stimuli and engaging them in a calming virtual environment. | Pain management,  Anxiety and stress reduction, | **Clinic** | Palliative care and Survivorship | Participants occasionally complained of dizziness associated with the goggles. |
| (Horesh et al., 2022) | VR | head-mounted display with headphones | not specified | two immersive scenarios: "Ocean Rift," a deep-sea diving experience, and "Happy Place," a tranquil beach setting. | Pain management,  Anxiety and stress reduction | **Clinic** | Palliative care and Survivorship | Cost of Equipment:  Feasibility for Home Use. |
| (Wilson & Scorsone, 2021) | VR | Xbox 360 Kinect™ Console (Microsoft) Games (Kinect sports, Fruit Ninja, Dance central) | not specified | The VR intervention included playing Kinect Sports I (darts, bowling, boxing) and Kinect Sports I (beach volleyball, table tennis), and Fruit Ninja for 30 minutes | Quality of life, Pain management | **Clinic** | Treatment | Cost of Equipment: The VR equipment was relatively expensive. |
| (Fabi et al., 2022) | VR | 3D visual aids, simulation, and interactive components | Virtual Environment for Radiotherapy Training (VERT) system. | It uses realistic 3D visualizations and interactive simulations to demonstrate patient anatomy, radiation doses, and the entire radiotherapy process, including immobilization, planning, and treatment delivery. | Education and Information | **Clinic** | Treatment | Resource Intensity |
| (Fahiminia et al., 2019) | VR | Nintendo Wii®. Siesta sleep system from Compumedics | Not mentioned | Mindfulness therapy to help minimize anxiety among patients | Anxiety and Stress Reduction | **Home** | Palliative care and survivorship | Visual disorders:  Technological Accessibility and Compliance |
| (Reynolds et al., 2022) | VR | VR One headet Glasses and headphones Phones | Not mentioned | The VR simulation featured local Florida scenes, including trees, water features, creeks, animals, and local parks. Nature sounds of chirping birds and running water were added to enhance the relaxation effect. | Anxiety and stress reduction | **Clinic** | Palliative care and survivorship | Not specified |
| (Park et al., 2023) | AR | Xbox Kinect Games included: darts, bowling, boxing, table tennis, Fruit Ninja, beach volleyball, and the “Macarena” dance for warm-up | UNICARE Home + | The games included activities like darts, bowling, boxing, table tennis, fruit ninja, and beach volleyball, all designed to promote active upper limb movements. | Rehabilitation and Physical Therapy, Improved Quality of Life | **Clinic** | Palliative Care and survivorship | Not specified |
| (Song et al., 2022) | VR | HTC VIVE (HTC Corporation, version VIVE pro2, Taiwan, China) system, which included virtual simulations of RT equipment. | Virtual Reality Radiotherapy (VRRT) | This system included visual and auditory elements that replicated the RT environment and procedures, aiming to reduce anxiety and improve understanding. Patients could virtually experience the RT process, which included viewing the treatment room, equipment, and the steps involved in RT | Education and Information | **Clinic** | Treatment | Not specified |
| (Garrett et al., 2020) | VR | Virtual Reality Glasses: Bluebee™ Genuine VR 3D Glasses provided by Faitem Plus S.L., Barcelona, Spain. | VR App | The tool provides a realistic virtual environment where patients can experience the various stages of their surgical admission process. The experience lasts 16 minutes and 34 seconds and covers steps from the initial interview with the surgeon, admission to the surgical ward, the operating room, and the postoperative recovery room. | Anxiety and Stress Reduction, Education and Information: | **Both** | Treatment | Not specified |
| (Chuan et al., 2023) | VR | Xbox 360 Kinect System by Microsoft, Redmond, WA, USA Your Shape Fitness Evolved 2012, Sport and Adventure games for the Xbox Kinect | Exergaming | The tool is an interactive home-based exercise program. This system leverages the Xbox 360 Kinect's motion-sensing capabilities to provide a variety of aerobic and strength exercises designed to improve physical fitness. | Rehabilitation and Physical Therapy | **Home** | Treatment | For home interventions, it is more challenging to supervise the patients while doing their exercise |
| (King et al., 2023) | VR | head-mounted glasses and two controllers. | VR-CALM | VR-CALM (Managing Cancer and Living Meaningfully based on VR), is a virtual reality-based psychotherapy designed to manage symptoms of cancer, relieve psychological distress, and improve the quality of life in breast cancer survivors. It immerses patients in a calming virtual environment, such as a beach house or Butterfly Valley, while they listen to ambient sounds and receive guided instructions from a CALM therapist. The therapy focuses on four areas: symptom management and health guidance, analysis of illness-related changes in self and relationships, exploration of life's meaning and purpose, and discussions about the future and hope. | Anxiety and stress reduction, Education and information, Improved quality of life, Symptom tracking and management | **Home** | Diagnosis | There was an acknowledgment of discomfort caused by wearing VR devices, including headaches, eyestrain, and nausea, which could affect user acceptance and continued use. |
| (Cartujano-Barrera et al., 2020) | VR | VR headset HTC VIVE and the free VR software Google Earth VR | simulated travel session using VR | The VR intervention allowed the patients to enjoy a 30-minute virtual travel session to memorable or desired locations. | Symptom Tracking and Management | **Clinic** | Palliative care and survivorship | Physical Discomfort: Some participants experienced mild discomfort, such as arm tiredness due to continuous operation, and motion sickness, including dizziness and nausea. Technical and Operational Challenges: Participants needed assistance from medical staff to operate the VR software, suggesting that the technology's complexity could be a barrier for some users. Environmental Conditions: Standardizing VR travel session time and conditions, including the presence of medical staff and family members, was noted as necessary, indicating variability in these factors as a potential barrier. |
| (Turrado et al., 2021) | VR | Head mounted display | N/A | The study used an immersive VR environment to simulate relaxing scenarios | Anxiety and stress reduction | **Clinic** | Treatment | N/A |
| (Torres García et al., 2023) | VR | Head‐mounted glasses (Vuzix Wrap 1200 VR) with a head motion tracking system and controllers | N/A | The VR tool provided a 3D immersive environment combined with music therapy for distraction | Anxiety and stress reduction | **Clinic** | Treatment | N/A |
| (Ashley Verzwyvelt et al., 2021) | VR | HTC VIVE Pro2.0 device | N/A | The VR system provides green therapy through VR to manage anxiety | Anxiety and stress reduction | **Clinic** | Treatment | Some patients showed discomfort of the oculomotor nerve and fatigue |
| (Burrai et al., 2023) | VR | HTC VIVE® VR headset | N/A | Immersive VR environment with a sunny landscape, waterfall flowing from snowy mountains into a lake, surrounded by a forest. Included a wooden house with a wharf to the lake, and seaside behind the house. Incorporated sounds of falling water and birds over a relaxing soundtrack. | Stress and anxiety reduction | **Clinic** | Treatment | N/A |
| (Mao et al., 2023) | VR | Google cardboard goggles | N/A | 3D side by side videos including Minion mini movies, Doraemon mini movie, and a green round object saving the town video | Stress and anxiety reduction | **Clinic** | Treatment | Limited field of view of Google cardboard goggles, holding the mobile phone uncomfortable; Parents concerned about excessive use causing dizziness and fatigue |
| (Schneider et al., 2011) | VR | Samsung Gear VR headset | N/A | Allows patients to play a selection of VR apps during chemotherapy. Apps include relaxation experiences and games | Anxiety and stress reduction, improved quality of life, pain management, | **Clinic** | Treatment | Patients did not experience any significant nausea from the VR, except in instances where the lenses could not be sufficiently adjusted for the appropriate focal length |
| (Zhao et al., 2023) | VR | Xbox 360 Kinect console | Your Shape Fitness Evolved 2012 game suite | Games used: "Wall Breaker," "Stomp It," and "Run the World" for upper and lower limb movements | Improved quality of life, rehabilitation and physical therapy | **Clinic** | Treatment | N/A |
| (Persson et al., 2021) | VR | Xbox360 Kinect console | Your Shape Fitness Evolved 2012 | Games used: "Wall Breaker," "Stomp It," and "Run the World" for upper and lower limb movements | Anxiety and Stress Reduction | **Clinic** | Palliative care and survivorship | N/A |
| (Schrempf et al., 2022) | VR | Nintendo Wii Fit Plus | N/A | A 6-week, home-based exercise intervention using light-intensity walking and balance exercises in a virtual reality environment. | Rehabilitation and physical therapy, improved quality of life, enhancing treatment adherence | **Home** | Treatment | N/A |
| (Jadmiko et al., 2022) | VR | Mirage Solo VR headset | Yuma's world | Interactive VR content with a basic three-level skill game in a calm underwater environment, including famous monuments and simple skill games. | Pain management, anxiety and stress reduction | **Home** | Palliative care and survivorship |  |
| (Baños et al., 2013) | VR | HTC VIVE Pro2.0 headset, a control handle, a positioning base station | VR rehabilitation management platform | The platform includes a head-mounted display, control handle, positioning base station, and laptop. It has patient, medical, and family interfaces with 13 Level one modules and 33 Level two modules | Enhanced treatment adherence, symptom tracking and management | **Both** | Palliative care and survivorship | Vertigo, safety concerns, economic cost, poor understanding of the system |
| (Cîmpean, 2019) | VR | Head-mounted display | N/A | The VR video included five chapters covering different aspects of a patient’s first day of IV chemotherapy, filmed from the patient’s point of view. | Education and information, stress and anxiety reduction | **Clinic** | Treatment | Not specified |
| (Niki et al., 2019) | VR | Head mounted display | N/A | The SafeSpace study developed a VR intervention incorporating compassionate mind training (CMT) to support people undergoing cancer treatment. The intervention included three short sessions focusing on relaxation and self-compassion exercises. | Anxiety and stress reduction, improved quality of life | **Clinic** | Palliative care and survivorship | Mild nausea and dizziness, external noise, equipment issues |
| (Tsuda et al., 2016) | VR | Oculus Quest headset | N/A | The VR tool provided a general overview of radiation oncology, specifically about the breast cancer RT process, including simulation, planning, daily treatment, and schedule. | Anxiety and stress reduction, education and information, Rehabilitation and Physical therapy, enhancing treatment adherence | **Clinic** | Treatment | Not specified |
| (Uslu & Arslan, 2023) | VR | Head-mounted display, specifically the Sony PC Glasstron PLM-S700. | virtual reality distraction intervention | The study explored the use of virtual reality (VR) distraction intervention to alleviate chemotherapy-related symptom distress in older women with breast cancer. Using a head-mounted display, the intervention provided immersive and interactive scenarios during chemotherapy sessions. The VR intervention showed a significant decrease in anxiety immediately following treatment, with a trend towards improved symptoms 48 hours after chemotherapy. Participants found the VR device easy to use, experienced no cybersickness, and expressed a willingness to use VR again. | Anxiety and stress reduction, Symptom Tracking and Management | Clinic | Treatment | From the patients' perspective, the barriers to XR use included concerns about the headset's weight and the quality of the visual image. |
| (Oyama et al., 1999) | VR | Mirage Solo VR headset by LENOVO S.r.l., which comes with a remote controller. Additionally, physiological signals were recorded using the Empatica E4 wristband. | Yuma’s World | The paper discusses a study on the use of Virtual Reality (VR) as a complementary therapy in home palliative care for advanced cancer patients. The VR intervention aimed to assess its effects on reducing anxiety, depression, and pain over four days and evaluate the immediate effects of VR sessions on cancer-related symptomatology. Participants were provided with a VR headset for four days, and their symptoms were measured before and after each VR session using various scales and physiological signal monitoring. The study found that while there were no significant changes in anxiety, depression, and pain over the four days, there was a significant improvement in symptoms immediately after the VR sessions. | Pain management, Anxiety and stress reduction, Symptom Tracking and Management, Symptom Tracking and Management | Home | Palliative care and Survivorship | Not mentioned |
| (Kaneda et al., 1999) | VR | VR glasses, a controller, and a screen, specifically implemented with "theBlu" Season 1 (Wevr, 2021) software for the VR session. | VR Blue | The paper discusses a pilot trial using a virtual reality (VR) intervention, VR Blue, designed to alleviate pain and related symptoms in patients with advanced stage colorectal cancer. The 30-minute VR session immersed patients in a calming underwater environment, aiming to assess feasibility, acceptability, safety, and impact on pain and distress. Results indicated high satisfaction, with significant improvements in pain, tension, stress, anxiety, and mood. No significant side effects were reported, supporting the safety of the intervention. | Pain management, Anxiety and stress reduction, Improved Quality of Life, Symptom Tracking and Management | Both | Treatment | Not mentioned |
| (Oyama et al., 2000) | VR | Bubble and Luna mobile applications, with the mobile phone placed inside designated VR goggles. | Bubble | The paper discusses a pilot study on an artificial intelligence–virtual reality (AI–VR) intervention named Bubble, designed to treat hot flashes and improve psychological well-being in women with breast and ovarian cancer. Bubble incorporates cognitive behavioral therapy and mindfulness-based stress reduction techniques within a VR environment called Frosty. | Anxiety and stress reduction, Improved Quality of Life, Symptom Tracking and Management | Home | Treatment | Not mentioned |
| (Schneider et al., 2004) | VR | VR headsets from the brand Applied VR. | Applied VR | The study explored the use of virtual reality (VR) to induce positive emotions and reduce anxiety in chemotherapy patients. It allowed participants to select a nature theme during treatment, aiming to shift mood positively and promote relaxation. The effectiveness was assessed by measuring mood and pain levels before and after the VR experience, along with participant satisfaction. Results indicated that VR helped participants feel more calm, relaxed, and content, although it did not significantly alter blood pressure or pain levels. Most participants preferred VR for future treatments, appreciating the immersive and distracting aspects of the experience. | Pain management, Anxiety and stress reduction | Clinic | Treatment | Not mentioned |
| (Schneider & Hood, 2007) | VR | VR headsets, specifically the Oculus Go headsets developed by Facebook Technologies in partnership with Qualcomm and Xiaomi. | Patient’s Dream | The "Patient's Dream" study explored the use of Virtual Reality Experience (VRE) as a distraction therapy during the first cycle of chemotherapy for patients with early breast and ovarian cancers. Assess the impact of VRE on psychological distress, anxiety, and quality of life. The results indicated that VRE could significantly reduce state anxiety and the perceived duration of chemotherapy treatment compared to conventional distraction methods. | Anxiety and stress reduction, Improved Quality of Life | Clinic | Treatment | Not mentioned |
| (Mao et al., 2024) | VR | VR headset and a large eye crystal capable of playing 3D film. The brands are not specified in the text. | Mindfulness Therapy by VR (Virtual-Reality) | The paper discusses the use of mindfulness therapy enhanced by virtual reality (VR) to alleviate death anxiety in a patient with cerebellar cancer. The therapy involved initial consultations and subsequent VR-assisted sessions at the patient's home, focusing on mindfulness practices. Significant improvements were observed in the patient's anxiety levels, blood pressure, heart rate, and body temperature, indicating the therapy's effectiveness in improving the patient's quality of life and psychological well-being. | Anxiety and stress reduction, Improved Quality of Life | Home | Treatment | depressive mood swings during the treatment, which were attributed to the chronic and dementia properties of the illness. . |
| (Hoffman et al., 2014) | VR | Not specified | Bedside Wellness System | Explore the use of immersive virtual reality (VR) as an adjunctive therapy for women with metastatic breast cancer (MBC) to alleviate physical and psychological symptoms. The study tested the acceptability and efficacy of VR interventions in improving quality of life and reducing fatigue, pain, depression, anxiety, and stress. Participants underwent two week-long VR experiences at home, with symptom assessments conducted before, after, and 48 hours post-intervention. Results indicated significant improvements in all measured outcomes, suggesting that VR is a feasible and acceptable home-based intervention that enhances the well-being of women with MBC. | Pain management, Anxiety and stress reduction, Improved Quality of Life, Symptom Tracking and Management | Home | Treatment | Not mentioned |
| BEC | AR (Augmented Reality) | UINCARE Home+ rehabilitation system and the Xbox One Kinect for Windows® motion capture device. The brands associated with these tools are UINCARE Corp. and Microsoft Corp., respectively. | UINCARE Home+ | The UINCARE Home+ system is an AR-based digital healthcare system designed for postoperative breast cancer patients to perform rehabilitation exercises at home. It uses the Xbox One Kinect to track movements in three-dimensional space, providing real-time feedback through visual and auditory cues to ensure exercises are performed correctly. The system delivers prescribed exercises directly to the patient's home, where they can follow along with on-screen instructions and receive instant feedback. This setup allows for remote monitoring and customization of the exercise program by physicians, enhancing the convenience and effectiveness of rehabilitation without the need for frequent hospital visits. | Pain management, Rehabilitation and Physical Therapy, Improved Quality of Life | Home | Palliative care and survivorship | Some patients described difficulties in using the system and complained of technical errors such as disconnection or connection delays, which affected their ability to use the system effectively. |
| (Glennon, 2018) | VR | Insta 360 Pro-I panoramic camera for capturing panoramic photos and the Pico Goblin VR all-in-one headset for displaying the VR environments. | Not mentioned | The study explores the psychophysiological recovery of cancer patients using VR to simulate natural environments. It involved 63 participants who viewed different types of landscapes (blue space, open green space, semi-open green space, closed green space, and gray space) through VR, assessing changes in psychological and physiological indicators before and after the sessions. | Anxiety and stress reduction | Clinic | Treatment | The challenges faced by patients while using the VR tool included discomfort that led to incomplete experimental data for some participants. |
| (Mohammad & Ahmad, 2019) | VR | HTC Vive stereoscopic headset running from a personal computer designed to support a high-quality VR experience. | Not mentioned | The paper discusses the use of virtual reality (VR) therapy as an adjunctive treatment for managing chronic cancer pain. It explores patients' experiences and perceptions through a qualitative study involving focus groups. The study found that while results were mixed, a majority of participants found VR therapy beneficial for pain management. VR provided a sense of immersion that helped distract from pain and facilitated relaxation. The study suggests that VR interventions should be designed with input from patients to maximize their efficacy and align with individual preferences. | Pain management | Home | Palliative care and survivorship | physical discomfort due to the weight of the headset, discomfort from wearing glasses inside the headset, and the need to sit or stand in uncomfortable positions for extended periods. |
| (Feyzioğlu et al., 2020) | VR | An Oculus Rift S virtual reality headset and hand-held controllers. The computer hardware specifications include a Core i7-8750H powered gaming laptop (Intel Corp, Mountain View, CA, USA), 16 gigabytes memory, and a dedicated multithread graphics core (GeForce GTX 1060, NVIDIA Corp, Santa Clara, CA, USA). | bespoke virtual reality-delivered pain therapy software programme | The paper discusses a bespoke virtual reality-delivered pain therapy software program designed to help cancer patients manage neuropathic pain. This program incorporates guided visualization and progressive muscle relaxation techniques, specifically tailored to minimize cybersickness in this vulnerable patient group. The pilot study evaluated the feasibility, acceptability, recruitment rates, and risk of cybersickness of the software, alongside secondary clinical outcomes like opioid consumption, pain severity, pain interference, and global quality of life scores. The results indicated that the virtual reality program was feasible and acceptable for cancer patients with neuropathic pain, showing trends towards reduced opioid use and pain severity in the intervention group compared to the control group. | Pain management, Improved Quality of Life, Rehabilitation and Physical Therapy | Clinic | Treatment | The challenges faced by patients while using the extended reality tool included experiencing cybersickness symptoms such as nausea, dizziness, disorientation, eyestrain, headaches, and fatigue. Additionally, pre-existing conditions like dizziness and nausea were significantly associated with an increased risk of cybersickness. |
| (Jimenez et al., 2018) | VR | Not specified | virtual reality relaxation (VR) intervention | The paper discusses a virtual reality (VR) intervention designed to alleviate distress and anxiety in patients with primary brain tumors (PBT) during clinical evaluations. The study, conducted from March 2021 to March 2022, involved a brief VR session prior to neuroimaging, with patient-reported outcomes collected before and after the intervention, and further assessments at 1 and 4 weeks. | Pain management, Anxiety and stress reduction, Psychological Support, Symptom Tracking and Management | Home | Treatment | The challenges faced by patients while using the VR tool included experiencing mild adverse effects such as headache, dizziness, nausea, and neck pain. |
| (Atef et al., 2020) | VR | 3D head-mounted virtual reality (VR) and 2D screen applications. The brands of the headsets are not specified in the text provided. | Not mentioned | The paper discusses the use of 3D head-mounted and 2D screen virtual reality (VR) applications to manage cancer pain in adults receiving palliative care. It highlights the feasibility and acceptability of these VR interventions, noting high completion rates and minimal side effects. Both VR formats were effective in reducing pain intensity, with 3D VR providing a higher sense of presence, which was associated with greater pain relief. | Pain management | Home | Palliative care and survivorship | Not mentioned |
| (Scates et al., 2020) | VR | virtual reality software and headsets, but the specific brands are not mentioned in the text provided. | Not mentioned | The paper discusses a clinical trial where virtual reality (VR) was used to reduce perioperative anxiety in patients undergoing colorectal cancer surgery. The VR software simulated all perioperative phases that the patient is awake for, from admission to discharge. The study measured anxiety levels using the State-Trait Anxiety Inventory Scale (STAI-S) and Hospital Anxiety and Depression Scale (HADS) before and after VR exposure. | Anxiety and stress reduction | Clinic | Treatment | Not mentioned |
| (Basha et al., 2022) | VR | Not specified | Not mentioned | The paper discusses a study that evaluated the effectiveness of a virtual reality intervention aimed at reducing anticipatory anxiety, depression, and emotional distress in breast cancer patients before their first chemotherapy dose. The study was comparative and randomized, involving two groups: one receiving virtual reality sessions and the other undergoing standard psychoeducational interventions based on mindfulness. | Anxiety and stress reduction, Education and Information, Rehabilitation and Physical therapy | Clinic | Palliative care and survivorship | Not mentioned |
| (Gao et al., 2022) | VR | Not specified | Green Therapy and Virtual Reality | The paper explores the use of Virtual Reality (VR) and Green Therapy (GT) in reducing pain and distress among oncology patients during chemotherapy sessions. The study involved 33 participants who experienced three different settings: a control room, a Green Therapy room, and a Virtual Reality room. Measurements of pain, distress, heart rate, blood pressure, and saliva cortisol levels were taken before and after the infusion in each room. Although the results did not show statistical significance in the reduction of these metrics, patients reported a more enjoyable experience in the VR and GT rooms compared to the control room. | Pain management, Anxiety and stress reduction | Clinic | Treatment | Not mentioned |
| (Turrado et al., 2021) | VR | Not specified | Not mentioned | The study evaluates the effectiveness of immersive Virtual Reality (VR) in reducing anxiety, fatigue, and pain in cancer patients undergoing antiblastic therapy. It was a randomized controlled trial with three groups: a VR group, a narrative medicine group, and a standard care group. The results indicated that VR significantly reduced anxiety and fatigue compared to the other groups, although it did not significantly affect pain levels. | Pain management, Anxiety and stress reduction | Clinic | Treatment | Not mentioned |
| (Villumsen et al., 2019) | VR | Pico Neo4 VR headset and somatosensory grips, manufactured by ByteDance. | Mindfulness Training Virtual Reality (MTVR) model | The virtual reality-based mindfulness training model for ovarian cancer patients undergoing chemotherapy includes four functional modules: personalized curriculum, intelligent monitoring, emotion tracking, and Funny Games. This model is designed to reduce anxiety, depression, and cancer-related fatigue. It offers a high level of acceptance among patients, significantly reducing psychological issues and improving patient well-being during chemotherapy. | Anxiety and stress reduction, Psychological Support | Home | Palliative care and survivorship | Not mentioned |
| (Zhang et al., 2022) | VR | head-mounted displays (HMDs), specifically the Sony PC Glasstron PLM-S700 and the iO Display Systems Inc. i-Glasses SVGA 3D. | virtual reality (VR). | The paper explores the use of virtual reality (VR) as a noninvasive intervention to make chemotherapy treatments more tolerable by altering patients' perception of time. VR helps patients perceive that time passes more quickly during treatment by distracting them from the chemotherapy environment. This effect, known as time compression, was particularly noted in patients with breast and colon cancer. The study used a crossover design with 137 participants, employing VR during one of their chemotherapy sessions and measuring the difference between the actual time elapsed and the patient's retrospective estimate of time. | Anxiety and stress reduction | Clinic | Treatment | not all patients can sustain full concentration for the duration of the chemotherapy session, which typically lasts 45-90 minutes and may require several hours. This suggests that maintaining concentration and engagement with the VR environment for extended periods could be challenging for some patients. |
| (Niki et al., 2019) | VR | VR glasses and a handheld controller. The brands of these tools are not specified in the text. | VR educational tool | VR glasses, a handheld controller, and provides an immersive experience with scientific knowledge of radiotherapy, a demonstration of the radiotherapy process, and an audio introduction. | Anxiety and stress reduction, Education and Information | Clinic | Palliative care and survivorship | Not mentioned |
| (Chirico et al., 2016) | VR | HTC Vive VR system, which includes a head-mounted display (HMD) and two hand controllers for manual interaction with the displayed environment. | Mysic Therapy | The paper discusses a virtual smash room designed for cancer rehabilitation, allowing patients to vent frustrations by breaking virtual objects like porcelain and glass items. This VR environment was tested by patients through a participatory design process, assessing its enjoyment and potential side effects like cybersickness. The study found the experience enjoyable and engaging, though some reported dizziness and difficulties with the interface. Suggestions for other VR environments were also gathered, indicating a broader potential application of VR in cancer rehabilitation. | Anxiety and Stress Reduction | Clinic | Treatment | The challenges faced by patients while using the virtual smash room tool included experiencing minor symptoms such as general discomfort, fatigue, nausea, and tired eyes. |
| (Chirico et al., 2020) | VR | Not specified | Not mentioned | The paper discusses a pilot study on the use of virtual reality (VR) for enhancing well-being, mood, and quality of life in cancer patients undergoing surgery. The study compared the effects of a VR-based intervention with a music-listening intervention and a control group receiving standard treatment. The VR intervention involved patients receiving VR sessions twice daily, which significantly reduced heart rate and respiratory rate, and improved overall mood more effectively than the music intervention. | Anxiety and stress reduction, Improved Quality of Life | Clinic | Treatment | Not mentioned |
| (Zhou et al., 2021) | VR | VR headsets, but the specific brands are not mentioned in the text provided. | My Comfortable Environment | The paper discusses a study on the impact of virtual reality (VR) content, "My Comfortable Environment," on the comfort levels of Indonesian cancer patients. This VR intervention, based on Kolcaba's comfort theory, was tested using a quasi-experimental design on 60 patients. The VR content included rural natural scenery, educational media, music therapy, and light activities like archery. Results indicated significant improvements in comfort levels post-intervention in the VR group compared to the control group, which received guided imagery. | Education and Information. Rehabilitation and physical therapy | Clinic | Palliative care and survivorship | Some obstacles while conducting an intervention were that the respondent needed time to adapt to the sophistication of VR technology for the first time, meaning that the researcher introduced the subjects to VR before the study commenced. |
| (Ioannou et al., 2022) | VR | A 32-inch LCD television connected to a computer, both installed on a trolley for mobility. Interaction devices used were a keyboard and mouse, and participants used headphones. | Mood Induction Procedure (MIP) using Virtual Reality (VR) | The paper discusses a pilot study on the feasibility and benefits of a virtual reality-based psychological intervention aimed at inducing positive emotions in hospitalized adult patients with advanced metastatic cancer. The intervention involved four 30-minute sessions over one week, where patients navigated virtual environments designed to induce joy or relaxation. The study assessed mood changes, patient satisfaction, and perceived utility of the intervention. Results indicated that the intervention was feasible, with no major difficulties in using the devices, and it was well-received by patients, providing benefits such as distraction, entertainment, and relaxation. Mood assessments showed an increase in positive emotions and a decrease in negative emotions. The intervention was considered minimally uncomfortable, suggesting its potential for wider implementation in similar medical settings. | Psychological Support | Clinic | Treatment | Navigation restrictions and limited interaction with the virtual environments, as reported by two users. Physical discomfort due to the posture required during the sessions, especially for those lying in bed, which made mouse movements difficult and provoked involuntary keystrokes. Disturbances during sessions from external factors such as visits from relatives, hospital staff entering the room, telephone calls, and roommates watching TV. Tiredness related to the uncomfortable position during the session, |
| (Janssen et al., 2022) | VR | The HTC VIVE headset and the free VR software Google Earth VR. | Google Earth VR | The study explores the use of virtual reality (VR) to simulate travel experiences for terminal cancer patients, aiming to alleviate their symptoms and fulfill their desires to visit memorable places. Utilizing Google Earth VR, the study demonstrated significant improvements in symptoms such as pain, tiredness, drowsiness, shortness of breath, depression, anxiety, and overall well-being, without serious side effects. The VR sessions were adaptable to patient preferences in duration and were facilitated with medical staff assistance. This approach suggests a promising palliative care tool for enhancing the quality of life in terminal cancer patients. | Pain management, Anxiety and stress reduction, Improved Quality of Life, Symptom Tracking and Management | Home | Treatment | No participants complained of serious side effects from VR travel. However, there were a few participants who reported increased ESAS scores after VR travel, indicating some discomfort or worsening of symptoms such as tiredness due to continuous operation, unknown reasons for increased drowsiness, nausea from the length of the experience, and dizziness due to ease of getting motion sickness. |
| (da Silva Alves et al., 2018) | VR | The Nintendo Wii Fit and the Wii Balance Board, both from Nintendo, Kyoto, Japan. | Nintendo Wii Fit | The study explored the feasibility and safety of a virtual reality exercise program using Nintendo Wii Fit for elderly patients with hematologic malignancies undergoing chemotherapy. The intervention involved daily 20-minute sessions of virtual reality exercises, five times a week, from the start of chemotherapy until hospital discharge | Anxiety and stress reduction | Both | Treatment | experiencing infections, tumor lysis syndrome, progression of the disease, and withdrawal of consent, which led to a high dropout rate from the study. Additionally, exercise sessions could not be performed on some days due to the unavailability of physical therapists. |
| (da Silva Alves et al., 2017) | VR | Not specified | Exergaming | The study investigates the impact of using virtual reality glasses on anxiety and fatigue among women with breast cancer undergoing adjuvant chemotherapy. Participants in the intervention group used VR glasses to watch and listen to beach and nature content for 30 minutes, while the control group received standard care. The study utilized the State Anxiety Scale and the Cancer Fatigue Scale for assessment before and after the intervention across four chemotherapy cycles. Results indicated that the intervention group experienced significant reductions in anxiety and fatigue scores compared to the control group, suggesting that VR glasses are an effective tool for alleviating these symptoms in breast cancer patients. | Anxiety and stress reduction, Symptom Tracking and Management | Both | Treatment | Not mentioned |
| (Hoffman et al., 2013) | VR | the virtual forest walk system includes a wide 3-screen LCD (liquid crystal display) with stereo sound, a walking system for the bedridden, and a scent system with a gentle breeze. The brands or specific manufacturers of these tools are not mentioned in the text. | Bedside Wellness System | The Bedside Wellness System, a virtual forest walk system, is designed to improve the emotional well-being of bedridden cancer patients by using virtual reality (VR) technology. It features a wide 3-screen LCD with stereo sound, a walking system for the bedridden, a scent system with a gentle breeze, and a system for monitoring vital signs. The system offers different scenic scenarios such as a park, a plateau, or a cherry blossom avenue. It aims to reduce anxiety, pain, and stress while increasing relaxation and emotional positivity. The system has shown potential in increasing pain thresholds, reducing stress levels, and enhancing patient satisfaction. Further improvements are needed to make the experience more natural and to allow patients more control over their virtual environment. | Pain management, Anxiety and stress reduction | Home18 to | Palliative care and Survivorship | The challenges faced by patients while using the virtual forest walk system included experiencing fatigue (with 1 patient being fairly fatigued and 4 a little fatigued) and the need for the system to be more natural and allow patients more control over their virtual environment to make the experience feel more natural and allow patients to feel that they are controlling where they walk |
| (Giannelli et al., 2024) | VR | the Virtual I-glasses Personal Display System (HMD) from Virtual I.O. inc., connected with RCA jacks to a Sony GV-UX7 NYSC video cassette recorder. | Virtual Forest Walk System: Bedside Wellness System (BSW) | The paper discusses the use of Virtual Reality Intervention Therapy (VRIT) to treat anticipatory nausea and vomiting in cancer patients undergoing chemotherapy. The therapy utilizes a head-mounted display (HMD) system to immerse patients in a virtual environment, providing a distraction from the negative anticipatory symptoms associated with chemotherapy. The study found that VRIT effectively reduced nausea and vomiting, decreased anxiety levels, and required less effort from patients compared to other behavioral interventions. The VR content, including movies, played a significant role in altering patients' cognitive states and enhancing the immersive experience. | Anxiety and stress reduction, Symptom Tracking and Management | Clinic | Treatment | Not mentioned |
| (Zhou et al., 2023) | VR | HTC VIVE Pro2.0 headset, a control handle, a positioning base station | VR rehabilitation management platform | The platform includes a head-mounted display, control handle, positioning base station, and laptop. It has patient, medical, and family interfaces with 13 Level one modules and 33 Level two modules. | Rehabilitation and physical therapy, enhanced treatment adherence, symptom tracking and management, family and caregiver support | Both | Palliative care and survivorship | Not mentioned |
| (Birkhoff, 2021) | VR | Head-mounted display | N/A | The VR video included five chapters covering different aspects of a patient’s first day of IV chemotherapy, filmed from the patient’s point of view. | Education and information, stress and anxiety reduction | Clinic | Treatment | Not mentioned |
| (O'Gara et al., 2022) | VR | Head-mounted display | N/A | The SafeSpace study developed a VR intervention incorporating compassionate mind training (CMT) to support people undergoing cancer treatment. The intervention included three short sessions focusing on relaxation and self-compassion exercises. | Psychological support, improved quality of life, stress and anxiety reduction | Clinic | Treatment | The barriers to XR use from the patients' perspective included the fact that despite finding the VR headset easy to use and experiencing no cybersickness, VR did not significantly decrease symptom distress either immediately or two days following chemotherapy treatments. |
| (Shin et al., 2023) | VR | Oculus Quest headset | N/A | The VR tool provided a general overview of radiation oncology, specifically of the breast cancer RT process, including simulation, planning, daily treatment, and schedule. | Stress and anxiety reduction, education and information | Clinic | Treatment | Not mentioned |

**Appendix 5 Measures used to evaluate the interventions**

| **Variable** | **Measure** | **Psychological or perceptional** | **Observational or physiological** | **Citation** |
| --- | --- | --- | --- | --- |
| Psychological distress and anxiety | Symptom Distress Scale (SDS) | X |  | (L. Zhang et al., 2022) |
|  | Depression Anxiety Stress Scales (DASS) | X |  | (Chuan et al., 2023; Reynolds et al., 2022) |
|  | EuroQoL EQ-D5_Anxiety/Depression | X |  | (House et al., 2016) |
|  | Hospital Anxiety and Depression Scale (HADS) | X |  | (Cîmpean, 2019; Fabi et al., 2022; Giannelli et al., 2024; Mao et al., 2023; Moscato et al., 2021; Oyama et al., 2000; Oyama et al., 1999; Torres García et al., 2023; Tsuda et al., 2016; Turrado et al., 2021) |
|  | Beck Anxiety Inventory (BDI_A) | X |  | (Fahiminia et al., 2019; Mao et al., 2024) |
|  | State Anxiety Inventory (SAI) | X |  | (Schneider et al., 2003; Schneider & Hood, 2007; Schneider et al., 2004; Uslu & Arslan, 2023) |
|  | State-Trait Anxiety Inventory (STAI) | X |  | (Birkhoff, 2021; Burrai et al., 2023; Chirico et al., 2020; Fabi et al., 2022; King et al., 2023; Schneider et al., 2011; Shin et al., 2023; Turrado et al., 2021; Wilson & Scorsone, 2021) |
|  | Amsterdam preoperative anxiety and information scale, APAIS) | X |  | (Shin et al., 2023; Zhao et al., 2023) |
|  | Kessler Psychological Distress Scale | X |  | (Horesh et al., 2022) |
|  | Self-Rating Depression Scale (SDS) | X |  | (Song et al., 2022) |
|  | Distress DME Scale | X |  | (Torres García et al., 2023) |
|  | UCLA Loneliness Scale | X |  | (King et al., 2023) |
|  | NCCN Distress thermometer | X |  | (King et al., 2023) |
|  | DNM-20 Adjustment Disorder | X |  | (King et al., 2023) |
|  | The Houston Methodist Cancer Service’s Distress Screening Assessment Tool | X |  | (Ashley Verzwyvelt et al., 2021) |
| Fatigue | Revised Piper Fatigue Scale (PFS) | X |  | (Burrai et al., 2023; Hoffman et al., 2013; Schneider et al., 2003; Schneider & Hood, 2007; Schneider et al., 2011; Schneider et al., 2004) |
|  | The Functional Assessment of Chronic Illness Therapy – Fatigue Scale (FACIT-Fatigue) | X |  | (da Silva Alves et al., 2018; da Silva Alves et al., 2017; Reynolds et al., 2022) |
|  | Cancer Related Fatigue Scale (CRF) | X |  | (Hoffman et al., 2013; Mao et al., 2023) |
|  | Cancer Fatigue Scale | X |  | (Oyama et al., 2000; Uslu & Arslan, 2023) |
| Pain | Brief Pain Inventory (BPI) | X |  | (Giannelli et al., 2024; Kelleher et al., 2022; Moscato et al., 2021; Reynolds et al., 2022) |
|  | Modified Brief Pain Inventory (mBPI) | X |  | (Chuan et al., 2023) |
|  | Self-reported version of the Leeds Assessment of  Neuropathic Symptoms and Signs (S-LANSS) | X |  | (Chuan et al., 2023) |
|  | The Houston Methodist Cancer Service’s Pain Assessment Tool | X |  | (Ashley Verzwyvelt et al., 2021) |
|  | Pain Numerical Rating Scale (NRS) | X |  | (Austin et al., 2022; Park et al., 2023) |
|  | Visual Analogue Scale (VAS) for pain intensiry/tolerance | X |  | (Burrai et al., 2023; Cîmpean, 2019; Garrett et al., 2020; Kelleher et al., 2022; Oyama et al., 1999) |
|  | McGill Pain Questionnaire (Weekly neuropathic pain) | X |  | (Garrett et al., 2020) |
|  | Pain severity | X |  | (Garrett et al., 2020; Schneider et al., 2003) |
|  | Pain Catastrophizing Scale (PCS) | X |  | (Chuan et al., 2023; Cîmpean, 2019) |
|  | Single oral morphine equivalent daily dose (oMEDD) for Opioid consumption |  | X | (Chuan et al., 2023) |
|  | Wong-baker scale | X |  | (Wilson & Scorsone, 2021) |
|  | Perceived Self-Efficacy for Fatigue Self-Management scale | X |  | (Hoffman et al., 2013) |
|  | EuroQoL EQ-D5_Pain/Discomfort | X |  | (Park et al., 2023; Reynolds et al., 2022) |
|  | Chronic Pain Self-Efficacy Scale | X |  | (Kelleher et al., 2022) |
| Quality of Life, technology acceptance, and Functional Outcomes | World Health Organization Quality of Life Scale-Brief Version | X |  | (Horesh et al., 2022) |
|  | Quality of Life EORTC_QOL | X |  | (Fabi et al., 2022; Schrempf et al., 2022) |
|  | Functional Assessment of Cancer Therapy-General Scale (FACT-G) | X |  | (Cîmpean, 2019; Ioannou et al., 2022) |
|  | Hand questionnaire [QuickDASH] score | X |  | (Park et al., 2023) |
|  | Disabilities of the Aerm shoulder |  | X | (Park et al., 2023) |
|  | Health Survey Short Form (SF-12) | X |  | (Garrett et al., 2020) |
|  | Shortened General Comfort Questionnaire | X |  | (Jadmiko et al., 2022) |
|  | Technology acceptance model (TAM) | X |  | (Mao et al., 2023) |
|  | Evaluation of VR intervention | X |  | (Schneider et al., 2004) |
|  | VR Participant Satisfaction questionnaire | X |  | (Burrai et al., 2023) |
|  | Was It Worth It (WIWI) | X |  | (King et al., 2023) |
|  | Linear Analogue Scale Assessment (LASA) | X |  | (Shin et al., 2023) |
|  | User Experience Themes | X |  | (Zhou et al., 2023) |
|  | Satisfaction | X |  | (Wilson & Scorsone, 2021) |
|  | Acceptance and Action Questionnaire | X |  | (O'Gara et al., 2022) |
|  | Satisfaction with Intervention Scale | X |  | (Baños et al., 2013) |
|  | Cancer Quality of Life Questionnaire-Core (QLQ-C30) | X |  | (Chuan et al., 2023; O'Gara et al., 2022) |
|  | Quality of life (Functional Assessment of Cancer Therapy-Breast [FACT-B] | X |  | (Park et al., 2023) |
|  | EuroQoL_5 | X |  | (Park et al., 2023; Reynolds et al., 2022) |
|  | System Usability Scale (SUS) | X |  | (Zhou et al., 2021) |
|  | Ease of use and Engagement |  | X | (Wong et al., 2022) |
| Mood and Psychological Well-being | Positive and Negative Affect Scale (PANAS) | X |  | (Janssen et al., 2022) |
|  | Self-Compassion Scale | X |  | (Wong et al., 2022) |
|  | Visual Analog Scale for Mood | X |  | (Song et al., 2022) |
|  | Overall mood | X |  | (O'Gara et al., 2022) |
|  | Profile of Mood States (POMS) | X |  | (Chirico et al., 2020; Ioannou et al., 2022; O'Gara et al., 2022) |
| Perceived Stress and Psychological Impact | Perceived Stress Scale | X |  | (Horesh et al., 2022) |
|  | Brief Illness Perception Questionnaire | X |  | (Horesh et al., 2022) |
|  | Cancer Behavior Inventory–Brief Version (CBI-B) | X |  | (Birkhoff, 2021) |
|  | Warwick and Edinburgh Mental Well-being Scale | X |  | (O'Gara et al., 2022) |
|  | Perceived Restorativeness Scale (PRS) | X |  | (Song et al., 2022) |
| Physical, ergonomic, and Physiological Measures  Symptom Management | Electrodermal activity (EDA) |  | X | (Moscato et al., 2021; O'Gara et al., 2022) |
|  | Heart rate (HR) |  | X | (Ashley Verzwyvelt et al., 2021; Birkhoff, 2021; Fahiminia et al., 2019; Ioannou et al., 2022; Moscato et al., 2021; O'Gara et al., 2022; Schrempf et al., 2022; Song et al., 2022; Wilson & Scorsone, 2021; Wong et al., 2022) |
|  | Pulse Rate (PR) |  | X | (Shin et al., 2023) |
|  | Skin temperature (SKT) OR body temperature (BT) |  | X | (Fahiminia et al., 2019; Moscato et al., 2021) |
|  | Activity Index (AI) |  | X | (Moscato et al., 2021) |
|  | Blood pressure (BP) |  | X | (Ashley Verzwyvelt et al., 2021; Birkhoff, 2021; Fahiminia et al., 2019; Ioannou et al., 2022; Oyama et al., 1999; Schrempf et al., 2022; Shin et al., 2023; Song et al., 2022; Wilson & Scorsone, 2021; Wong et al., 2022) |
|  | Muscle Strength |  | X | (Wong et al., 2022) |
|  | Surface electromyography (sEMG) |  | X | (da Silva Alves et al., 2017) |
|  | Change in range of motion (ROM) of the affected shoulder |  | X | (da Silva Alves et al., 2017) |
|  | Common Terminology Criteria for Adverse Events (CTCAE) |  | X | (Park et al., 2023) |
|  | iGroup Presence Questionnaire (IPQ) | X |  | (Fabi et al., 2022; Tsuda et al., 2016) |
|  | Subjective feelings | X |  | (Austin et al., 2022) |
|  | Nausea levels | X |  | (Oyama et al., 1999) |
|  | Oxygen Saturation |  | X | (Ioannou et al., 2022) |
|  | Electroencephalogram (EEG) |  | X | (Song et al., 2022) |
|  | Neutrophil-to-lymphocyte ratio (NLR) |  | X | (Song et al., 2022) |
|  | Respiratory rate |  | X | (Oyama et al., 1999; Schrempf et al., 2022; Shin et al., 2023) |
|  | Saliva cortisol levels |  | X | (Ashley Verzwyvelt et al., 2021) |
|  | Walking duration and balance | X |  | (Hoffman et al., 2013) |
|  | Blood flow volume under a fingernail |  | X | (Oyama et al., 1999) |
|  | Frequency of vomiting |  | X | (Kaneda et al., 1999) |
|  | Barthel index (independency in mobility and physical fittness) |  | X | (Tsuda et al., 2016) |
|  | EuroQoL EQ-D5_Mobility | X |  | (Park et al., 2023; Reynolds et al., 2022) |
|  | Handgrip strength |  | X | (Tsuda et al., 2016) |
|  | Steps per day (measured by pedometer) |  | X | (Hoffman et al., 2013) |
|  | Knee extension strength |  | X | (Tsuda et al., 2016) |
|  | One-leg standing time |  | X | (Tsuda et al., 2016) |
|  | Set-up errors |  | X | (Zhao et al., 2023) |
|  | VR symptom questionnaire for side effects | X |  | (Burrai et al., 2023) |
|  | Edmonton Symptom Assessment Scale (ESAS) | X |  | (Austin et al., 2022; Giannelli et al., 2024; Janssen et al., 2022; Moscato et al., 2021; Niki et al., 2019) |
|  | Patient Reported Outcomes-CTCAE (PRO-CTCAE) | X |  | (Fabi et al., 2022; King et al., 2023) |
|  | Adapted Symptom Distress Scale | X |  | (Schneider & Hood, 2007) |
|  | Virtual Reality Symptoms Questionnaire (VRSQ) (general discomfort, fatigue, nausea, tired eyes, and dizziness) | X |  | (Chirico et al., 2020; Persson et al., 2021) |
|  | Simulator Sickness Questionnaire (SSQ) | X |  | (Zhou et al., 2021) |
| Sleep Quality | Pittsburgh Sleep Quality Index | X |  | (Garrett et al., 2020; Horesh et al., 2022) |
| Specific Condition-Related Measures | Hot Flash Related Daily Interference Scale | X |  | (Horesh et al., 2022) |
|  | Hot Flash Rating Scale | X |  | (Horesh et al., 2022) |
|  | Presence Questionnaire | X |  | (Schneider & Hood, 2007; Zhou et al., 2021) |
| Additional measures | Actual time elapsed during chemotherapy with VR intervention |  | X | (Fabi et al., 2022; Schneider et al., 2011) |
|  | Perceived time elapsed during chemotherapy with VR intervention as estimated by the patient | X |  | (Chirico et al., 2016; Fabi et al., 2022; Schneider et al., 2011) |
|  | Intrinsic Motivation Inventory (IMI) (interest/enjoyment and felt pressure and tension) | X |  | (Persson et al., 2021) |
|  | Visual Analog Scale for Physical Discomfort | X |  | (Baños et al., 2013) |
|  | Visual Analog Scale for Satisfaction | X |  | (Baños et al., 2013) |
|  | Australian-modifed Karnofsky Performance Status (AKPS) | X |  | (Austin et al., 2022) |
|  | Adherence rate |  | X | (Kelleher et al., 2022; Tsuda et al., 2016) |
|  | EuroQoL EQ-D5_Self-care | X |  | (Park et al., 2023; Reynolds et al., 2022) |
|  | MINI-MAC coping scale | X |  | (Torres García et al., 2023) |
|  | Coping Strategies Questionnaire (CSQ) | X |  | (Kelleher et al., 2022) |
|  | EuroQoL EQ-D5_Usual Activities | X |  | (Park et al., 2023; Reynolds et al., 2022) |
|  | Scores of the timed up and go test and Instrumental Activities of Daily Living |  | X | (Tsuda et al., 2016) |

**Appendix 6 Challenges of XR Use**

The studies and interventions highlighted several challenges worth noting as part of this review. The challenges involved physical discomfort and health concerns, technical and operational challenges, environmental and contextual challenges, resource and cost challenges, and user experience and compliance. More details on these challenges are in Appendix XX.

**Physical discomfort and health concerns**

Some patients experienced physical discomfort related to their health situations (Ashley Verzwyvelt et al., 2021; Cartujano-Barrera et al., 2020), such as discomfort of the oculomotor nerve, fatigue (Ashley Verzwyvelt et al., 2021), and tiredness of the arms (Cartujano-Barrera et al., 2020). Another adverse effect that patients faced was nausea, eyestrain, and headaches associated with the goggles, referred to in some other studies as motion sickness, vertigo, dizziness, disorientation, and cybersickness, especially in interventions involving movement (Baños et al., 2013; Cartujano-Barrera et al., 2020; Chirico et al., 2020; Feyzioğlu et al., 2020; Kelleher et al., 2022; King et al., 2023; Mao et al., 2024; Mohammad & Ahmad, 2019; Niki et al., 2019). Other minor symptoms were reported, such as fatigue, tired eyes, and neck pain (Chirico et al., 2016; Feyzioğlu et al., 2020; Jimenez et al., 2018). In one of the experiments, patients reported pain and discomfort due to posture while undergoing the experiment (Ioannou et al., 2022; Moscato et al., 2021). Some patients did not appreciate needing to sit or stand in uncomfortable positions for extended periods (Mohammad & Ahmad, 2019). While some challenges can be accounted for and addressed, others cannot. For example, some participants suffered from the progression of their diseases that resulted in withdrawal of consent during virtual reality exercise (da Silva Alves et al., 2018; da Silva Alves et al., 2017).

**Technical and operational challenges**

In addition to the physical discomfort-related challenges, some studies reported technical and operational challenges. For instance, some participants complained about the equipment issues (Ioannou et al., 2022; Niki et al., 2019). Some of the technical challenges came from occasional hardware issues like temporary controller failures and tripping over wires (Chirico et al., 2016) and from technical errors like disconnection or connection delays (House et al., 2016), and the quality of the images provided (Uslu & Arslan, 2023). Tools like Google cardboard goggles provided limited fields of view and made it uncomfortable to go through the experiments holding mobile phones (Mao et al., 2023, 2024). Some other challenges came from the discomfort caused by the weight of the headsets and the discomfort caused by having to wear glasses (Mohammad & Ahmad, 2019; Uslu & Arslan, 2023). Some participants also experienced some human-computer interaction issues. For instance, some needed assistance from medical staff to operate the VR software (Cartujano-Barrera et al., 2020), and other participants experienced difficulties in gripping virtual tools (Chirico et al., 2016; Chirico et al., 2020).

**Environmental, resource, cost, and contextual challenges**

Some problems mentioned in the studies were related to environmental and contextual challenges of the experiments conducted. For instance, some participants complained about the external noise and disturbances in the experimental environment (Cartujano-Barrera et al., 2020; Ioannou et al., 2022; Niki et al., 2019). While it is not always possible to provide the significant human resources required, some participants preferred the presence of medical staff and family members (Cartujano-Barrera et al., 2020; Fabi et al., 2022) with more supervision needs required for home interventions (Chuan et al., 2023; Fabi et al., 2022), and some complained about the unavailability of physical therapists for exercise sessions (da Silva Alves et al., 2018; da Silva Alves et al., 2017; Fabi et al., 2022). Furthermore, the high cost of the VR equipment was mentioned as one of the anticipated challenges to the success of the interventions of Horesh et al. and Wilson et al. (Horesh et al., 2022; Wilson & Scorsone, 2021). Finally, it is noteworthy that some participants complained about the home use of such technologies hindering them from accomplishing their daily goals and activities (Horesh et al., 2022).

**User experience and compliance challenges**

Some challenges were related to the user experience and compliance with the experiments. For instance, some patients experienced depressive mood swings and discomfort that resulted in incomplete experimental data (Glennon, 2018). Additionally, some patients had difficulty maintaining concentration and engagement with the VR environment for extended periods (X. Zhang et al., 2022). Because of discomfort and fatigue, it was also hard for some participants to comply with consistent use of the VR technology (Fahiminia et al., 2019; Hoffman et al., 2013; Hoffman et al., 2014). Furthermore, visual impairment and imagery preference issues affected the video game-based performance (Fahiminia et al., 2019; Hoffman et al., 2013; Hoffman et al., 2014). Finally, not all the studies prepared participants well before the start of the experiment. The study by Zhou et al. noted that one of the challenges mentioned by the participants was the need for adaptation to the sophistication of VR technology before the study commenced (Zhou et al., 2023; Zhou et al., 2021).

**Appendix 7. Study Limitations**

While this review has many strengths, it has some limitations worth acknowledging. First, we only included articles written in English. We may have missed relevant articles that are written in different languages. In addition, the XR technologies and interventions included in the study varied widely in terms of content, strategies, and specifications. This heterogeneity may complicate the interpretation of results and limit the ability to draw consistent conclusions about the effectiveness of XR technologies across different cancer care settings. Furthermore, the studies reviewed were conducted in various settings (clinic vs. home), and the technologies were tailored to different patient needs. This diversity may limit the generalizability of the findings to other contexts or patient groups, particularly where resources or access to technology are limited. We did not differentiate between research-developed tools and commercially available ones in the reporting of the findings, which can be covered in future research. Finally, this study focused on studying the use of XR across the different cancer continuum phases. Although we reported the type of cancer for each of the populations that the tools were designed for, it would be beneficial to compare the findings per type of cancer in future studies.

Based on the findings, we propose the following key directions for future research. First, a more targeted review examining XR interventions for specific cancer types (e.g., breast, lung, or hematologic malignancies) could provide deeper insights into cancer-specific symptom management and patient needs. Second, the majority of current XR interventions focus on treatment and survivorship. Future research should explore applications in: (1) Prevention and early detection (e.g., XR-based education and risk assessment tools), and (2) Diagnosis (e.g., XR-assisted imaging interpretation and biopsy simulations). Additionally, future studies should develop consistent intervention protocols, including: session length and frequency of XR use, optimal headset types and settings for different patient needs, and longitudinal follow-up studies to assess sustained impact over time. By addressing these research gaps, future studies can provide stronger evidence on the role of XR in oncology, ultimately improving patient outcomes and clinical implementation strategies.

Ashley Verzwyvelt, L., McNamara, A., Xu, X., & Stubbins, R. (2021). Effects of virtual reality v. biophilic environments on pain and distress in oncology patients: a case-crossover pilot study. *Scientific Reports*, *11*(1), 20196.

Atef, D., Elkeblawy, M. M., El-Sebaie, A., & Abouelnaga, W. A. I. (2020). A quasi-randomized clinical trial: virtual reality versus proprioceptive neuromuscular facilitation for postmastectomy lymphedema. *Journal of the Egyptian National Cancer Institute*, *32*, 1-9.

Austin, P. D., Siddall, P. J., & Lovell, M. R. (2022). Feasibility and acceptability of virtual reality for cancer pain in people receiving palliative care: a randomised cross-over study. *Supportive care in cancer*, *30*(5), 3995-4005.

Baños, R. M., Espinoza, M., García-Palacios, A., Cervera, J. M., Esquerdo, G., Barrajón, E., & Botella, C. (2013). A positive psychological intervention using virtual reality for patients with advanced cancer in a hospital setting: a pilot study to assess feasibility. *Supportive care in cancer*, *21*, 263-270.

Basha, M. A., Aboelnour, N. H., Alsharidah, A. S., & Kamel, F. H. (2022). Effect of exercise mode on physical function and quality of life in breast cancer–related lymphedema: a randomized trial. *Supportive care in cancer*, 1-10.

Birkhoff, S. D. (2021). The effects of virtual reality on anxiety and self-efficacy among patients with cancer: a pilot study. *Number 4/July 2021*, *48*(4), 431-439.

Burrai, F., Ortu, S., Marinucci, M., De Marinis, M. G., & Piredda, M. (2023). Effectiveness of immersive virtual reality in people with cancer undergoing antiblastic therapy: a randomized controlled trial. Seminars in Oncology Nursing,

Cartujano-Barrera, F., Sanderson Cox, L., Arana-Chicas, E., Ramírez, M., Perales-Puchalt, J., Valera, P., Díaz, F. J., Catley, D., Ellerbeck, E. F., & Cupertino, A. P. (2020). Feasibility and acceptability of a culturally-and linguistically-adapted smoking cessation text messaging intervention for Latino smokers. *Frontiers in Public Health*, *8*, 269.

Chan, R. J., Milch, V. E., Crawford‐Williams, F., Agbejule, O. A., Joseph, R., Johal, J., Dick, N., Wallen, M. P., Ratcliffe, J., & Agarwal, A. (2023). Patient navigation across the cancer care continuum: an overview of systematic reviews and emerging literature. *CA: a cancer journal for clinicians*, *73*(6), 565-589.

Chirico, A., D’Aiuto, M., Pinto, M., Milanese, C., Napoli, A., Avino, F., Iodice, G., Russo, G., De Laurentiis, M., & Ciliberto, G. (2016). The elapsed time during a virtual reality treatment for stressful procedures. A pool analysis on breast cancer patients during chemotherapy. Intelligent Interactive Multimedia Systems and Services 2016,

Chirico, A., Maiorano, P., Indovina, P., Milanese, C., Giordano, G. G., Alivernini, F., Iodice, G., Gallo, L., De Pietro, G., & Lucidi, F. (2020). Virtual reality and music therapy as distraction interventions to alleviate anxiety and improve mood states in breast cancer patients during chemotherapy. *Journal of cellular physiology*, *235*(6), 5353-5362.

Chuan, A., Hatty, M., Shelley, M., Lan, A., Chow, H., Dai, E., Haider, S., Bogdanovych, A., & Chua, W. (2023). Feasibility of virtual reality‐delivered pain psychology therapy for cancer‐related neuropathic pain: a pilot randomised controlled trial. *Anaesthesia*, *78*(4), 449-457.

Cîmpean, A. I. (2019). A pilot study to compare cognitive behavioral therapy with virtual reality vs. standard cognitive behavioral therapy for patients who suffer from cervical cancer. *Journal of Evidence-Based Psychotherapies*, *19*(1), 115-127.

da Silva Alves, R., Iunes, D. H., de Carvalho, J. M., Menezes, F. d. S., Silva, A. M., Borges, J. B. C., & Carvalho, L. C. (2018). Effects of exergaming on quality of life in cancer patients. *Games for Health Journal*, *7*(6), 385-392.

da Silva Alves, R., Iunes, D. H., Pereira, I. C., Borges, J. B. C., Nogueira, D. A., Silva, A. M., Lobato, D. F. M., & Carvalho, L. C. (2017). Influence of exergaming on the perception of cancer-related fatigue. *Games for Health Journal*, *6*(2), 119-126.

Fabi, A., Fotia, L., Giuseppini, F., Gaeta, A., Falcicchio, C., Giuliani, G., Savarese, A., Taraborelli, E., Rossi, V., & Malaguti, P. (2022). The immersive experience of virtual reality during chemotherapy in patients with early breast and ovarian cancers: The patient’s dream study. *Frontiers in Oncology*, *12*, 960387.

Fahiminia, S., Salahiyan, A., & Norouzi, M. (2019). The Effectiveness of Mindfulness Therapy by VR (Virtual-Reality) With a Focus on Death Anxiety in a Patient With Cerebellar Cancer (Case Report). *International Clinical Neuroscience Journal*, *6*(1), 33-35.

Feyzioğlu, Ö., Dinçer, S., Akan, A., & Algun, Z. C. (2020). Is Xbox 360 Kinect-based virtual reality training as effective as standard physiotherapy in patients undergoing breast cancer surgery? *Supportive Care in Cancer*, *28*, 4295-4303.

Garrett, B. M., Tao, G., Taverner, T., Cordingley, E., & Sun, C. (2020). Patients perceptions of virtual reality therapy in the management of chronic cancer pain. *Heliyon*, *6*(5).

Giannelli, A., Moscato, S., Ostan, R., Pannuti, R., Chiari, L., Biasco, G., & Varani, S. (2024). Virtual Reality for advanced cancer patients assisted at home: A randomized controlled interventional study. *Psycho‐Oncology*, *33*(7), e6368.

Glennon, C. (2018). Use of virtual reality to distract from pain and anxiety. *Number 4/July 2018*, *45*(4), 545-552.

Hoffman, A. J., Brintnall, R. A., Brown, J. K., Von Eye, A., Jones, L. W., Alderink, G., Ritz-Holland, D., Enter, M., Patzelt, L. H., & VanOtteren, G. M. (2013). Too sick not to exercise: using a 6-week, home-based exercise intervention for cancer-related fatigue self-management for postsurgical non–small cell lung cancer patients. *Cancer nursing*, *36*(3), 175-188.

Hoffman, A. J., Brintnall, R. A., Brown, J. K., Von Eye, A., Jones, L. W., Alderink, G., Ritz-Holland, D., Enter, M., Patzelt, L. H., & VanOtteren, G. M. (2014). Virtual reality bringing a new reality to postthoracotomy lung cancer patients via a home-based exercise intervention targeting fatigue while undergoing adjuvant treatment. *Cancer nursing*, *37*(1), 23-33.

Horesh, D., Kohavi, S., Shilony-Nalaboff, L., Rudich, N., Greenman, D., Feuerstein, J. S., & Abbasi, M. R. (2022). Virtual reality combined with artificial intelligence (vr-ai) reduces hot flashes and improves psychological well-being in women with breast and ovarian cancer: A pilot study. Healthcare,

House, G., Burdea, G., Grampurohit, N., Polistico, K., Roll, D., Damiani, F., Hundal, J., & Demesmin, D. (2016). A feasibility study to determine the benefits of upper extremity virtual rehabilitation therapy for coping with chronic pain post-cancer surgery. *British Journal of Pain*, *10*(4), 186-197.

Ioannou, A., Paikousis, L., Papastavrou, E., Avraamides, M. N., Astras, G., & Charalambous, A. (2022). Effectiveness of Virtual Reality Vs Guided Imagery on mood changes in cancer patients receiving chemotherapy treatment: A crossover trial. *European Journal of Oncology Nursing*, *61*, 102188.

Jadmiko, A. W., Kristina, T. N., Sujianto, U., Prajoko, Y. W., Dwiantoro, L., & Widodo, A. P. (2022). A Quasi-experimental of a Virtual Reality Content Intervention for Level of Comfort of Indonesian Cancer Patients. *CIN: Computers, Informatics, Nursing*, *40*(12), 841-847.

Janssen, A., Fletcher, J., Keep, M., Ahmadpour, N., Rouf, A., Marthick, M., & Booth, R. (2022). Experiences of patients undergoing chemotherapy with virtual reality: Mixed methods feasibility study. *JMIR serious games*, *10*(1), e29579.

Jimenez, Y. A., Cumming, S., Wang, W., Stuart, K., Thwaites, D. I., & Lewis, S. J. (2018). Patient education using virtual reality increases knowledge and positive experience for breast cancer patients undergoing radiation therapy. *Supportive care in cancer*, *26*, 2879-2888.

Kaneda, M., Oyama, H., & Katsumata, N. (1999). VR intervention therapy for emotion related cancer chemotherapy side effects. Int Conf Artif Real Telexistence,

Kelleher, S. A., Fisher, H. M., Winger, J. G., Miller, S. N., Amaden, G. H., Somers, T. J., Colloca, L., Uronis, H. E., & Keefe, F. J. (2022). Virtual reality for improving pain and pain-related symptoms in patients with advanced stage colorectal cancer: A pilot trial to test feasibility and acceptability. *Palliative & supportive care*, *20*(4), 471-481.

King, A. L., Roche, K. N., Leeper, H. E., Vera, E., Mendoza, T., Mentges, K., Acquaye-Mallory, A. A., Adegbesan, K. A., Boris, L., & Burton, E. (2023). Feasibility of a virtual reality intervention targeting distress and anxiety symptoms in patients with primary brain tumors: Interim analysis of a phase 2 clinical trial. *Journal of neuro-oncology*, *162*(1), 137-145.

Mao, W., Chen, W., & Wang, Y. (2023). Effect of virtual reality-based mindfulness training model on anxiety, depression, and cancer-related fatigue in ovarian cancer patients during chemotherapy. *Technology and Health Care*(Preprint), 1-14.

Mao, W., Chen, W., & Wang, Y. (2024). Effect of virtual reality-based mindfulness training model on anxiety, depression, and cancer-related fatigue in ovarian cancer patients during chemotherapy. *Technology and Health Care*(Preprint), 1-14.

Mohammad, E. B., & Ahmad, M. (2019). Virtual reality as a distraction technique for pain and anxiety among patients with breast cancer: a randomized control trial. *Palliative & supportive care*, *17*(1), 29-34.

Moscato, S., Sichi, V., Giannelli, A., Palumbo, P., Ostan, R., Varani, S., Pannuti, R., & Chiari, L. (2021). Virtual reality in home palliative care: brief report on the effect on cancer-related symptomatology. *Frontiers in Psychology*, *12*, 709154.

Niki, K., Okamoto, Y., Maeda, I., Mori, I., Ishii, R., Matsuda, Y., Takagi, T., & Uejima, E. (2019). A novel palliative care approach using virtual reality for improving various symptoms of terminal cancer patients: a preliminary prospective, multicenter study. *Journal of palliative medicine*, *22*(6), 702-707.

O'Gara, G., Murray, L., Georgopoulou, S., Anstiss, T., Macquarrie, A., Wheatstone, P., Bellman, B., Gilbert, P., Steed, A., & Wiseman, T. (2022). SafeSpace: what is the feasibility and acceptability of a codesigned virtual reality intervention, incorporating compassionate mind training, to support people undergoing cancer treatment in a clinical setting? *BMJ open*, *12*(2), e047626.

Oyama, H., Kaneda, M., Katsumata, N., Akechi, T., & Ohsuga, M. (2000). Using the bedside wellness system during chemotherapy decreases fatigue and emesis in cancer patients. *Journal of medical systems*, *24*, 173-182.

Oyama, H., Ohsuga, M., Tatsuno, Y., & Katsumata, N. (1999). Evaluation of the psycho-oncological effectiveness of the bedside wellness system. *CyberPsychology & Behavior*, *2*(1), 81-84.

Park, H.-Y., Nam, K. E., Lim, J.-Y., Yeo, S. M., Lee, J. I., & Hwang, J. H. (2023). Real-time interactive digital health care system for postoperative breast cancer patients: a randomized controlled trial. *Telemedicine and e-Health*, *29*(7), 1057-1067.

Persson, J., Clifford, D., Wallergård, M., & Sandén, U. (2021). A virtual smash room for venting frustration or just having fun: Participatory design of virtual environments in digitally reinforced cancer rehabilitation. *JMIR Rehabilitation and Assistive Technologies*, *8*(4), e29763.

Reynolds, L. M., Cavadino, A., Chin, S., Little, Z., Akroyd, A., Tennant, G., Dobson, R., Broom, R., & Gautier, A. (2022). The benefits and acceptability of virtual reality interventions for women with metastatic breast cancer in their homes; a pilot randomised trial. *BMC cancer*, *22*(1), 360.

Scates, D., Dickinson, J. I., Sullivan, K., Cline, H., & Balaraman, R. (2020). Using nature-inspired virtual reality as a distraction to reduce stress and pain among cancer patients. *Environment and Behavior*, *52*(8), 895-918.

Schneider, S. M., Ellis, M., Coombs, W. T., Shonkwiler, E. L., & Folsom, L. C. (2003). Virtual reality intervention for older women with breast cancer. *CyberPsychology & Behavior*, *6*(3), 301-307.

Schneider, S. M., & Hood, L. E. (2007). Virtual reality: a distraction intervention for chemotherapy. Oncology nursing forum,

Schneider, S. M., Kisby, C. K., & Flint, E. P. (2011). Effect of virtual reality on time perception in patients receiving chemotherapy. *Supportive care in cancer*, *19*, 555-564.

Schneider, S. M., Prince-Paul, M., Allen, M. J., Silverman, P., & Talaba, D. (2004). Virtual reality as a distraction intervention for women receiving chemotherapy. Oncology nursing forum,

Schrempf, M. C., Petzold, J., Petersen, M. A., Arndt, T. T., Schiele, S., Vachon, H., Vlasenko, D., Wolf, S., Anthuber, M., & Müller, G. (2022). A randomised pilot trial of virtual reality-based relaxation for enhancement of perioperative well-being, mood and quality of life. *Scientific Reports*, *12*(1), 12067.

Shin, J., Chang, J. S., Kim, J. S., An, J.-Y., Chung, S. Y., Yoon, S.-Y., & Kim, Y. B. (2023). An investigation of the effect of virtual reality on alleviating anxiety in patients with breast cancer undergoing radiation therapy: A randomized controlled trial. *International Journal of Radiation Oncology* Biology* Physics*, *117*(5), 1191-1199.

Song, R., Chen, Q., Zhang, Y., Jia, Q. a., He, H., Gao, T., & Qiu, L. (2022). Psychophysiological restorative potential in cancer patients by virtual reality (VR)-based perception of natural environment. *Frontiers in Psychology*, *13*, 1003497.

Torres García, A., Morcillo Serra, C., Argilés Huguet, M., González Gardó, L., Abad Esteve, A., & Ramos Quiroga, J. A. (2023). Efficacy of a Virtual Reality Intervention for Reducing Anxiety, Depression, and Increasing Disease Coping in Patients with Breast Cancer Before Their First Chemotherapy Dose. *Cognitive Therapy and Research*, 1-15.

Torres García, A., Morcillo Serra, C., Argilés Huguet, M., González Gardó, L., Abad Esteve, A., & Ramos Quiroga, J. A. (2024). Efficacy of a Virtual Reality Intervention for Reducing Anxiety, Depression, and Increasing Disease Coping in Patients with Breast Cancer Before Their First Chemotherapy Dose. *Cognitive Therapy and Research*, *48*(3), 451-465.

Tsuda, K., Sudo, K., Goto, G., Takai, M., Itokawa, T., Isshiki, T., Takei, N., Tanimoto, T., & Komatsu, T. (2016). A feasibility study of virtual reality exercise in elderly patients with hematologic malignancies receiving chemotherapy. *Internal Medicine*, *55*(4), 347-352.

Turrado, V., Guzmán, Y., Jiménez-Lillo, J., Villegas, E., de Lacy, F. B., Blanch, J., Balibrea, J. M., & Lacy, A. (2021). Exposure to virtual reality as a tool to reduce peri-operative anxiety in patients undergoing colorectal cancer surgery: a single-center prospective randomized clinical trial. *Surgical endoscopy*, *35*, 4042-4047.

Uslu, A., & Arslan, S. (2023). The Effect of Using Virtual Reality Glasses on Anxiety and Fatigue in Women with Breast Cancer Receiving Adjuvant Chemotherapy: A Pretest-Posttest Randomized Controlled Study. Seminars in Oncology Nursing,

Villumsen, B. R., Jorgensen, M. G., Frystyk, J., Hørdam, B., & Borre, M. (2019). Home‐based ‘exergaming’was safe and significantly improved 6‐min walking distance in patients with prostate cancer: a single‐blinded randomised controlled trial. *BJU international*, *124*(4), 600-608.

Wilson, K., & Scorsone, G. (2021). The use of virtual reality technologies to reduce anxiety and improve experience in chemotherapy patients during treatment. *Frontiers in Virtual Reality*, *2*, 695449.

Wong, C. L., Li, C. K., Choi, K. C., So, W. K. W., Kwok, J. Y. Y., Cheung, Y. T., & Chan, C. W. H. (2022). Effects of immersive virtual reality for managing anxiety, nausea and vomiting among paediatric cancer patients receiving their first chemotherapy: an exploratory randomised controlled trial. *European Journal of Oncology Nursing*, *61*, 102233.

Zhang, L., Liu, X., Tong, F., Zhou, R., Peng, W., Yang, H., Liu, F., Yang, D., Huang, X., & Wen, M. (2022). The prevalence of psychological disorders among cancer patients during the COVID‐19 pandemic: A meta‐analysis. *Psycho‐Oncology*, *31*(11), 1972-1987.

Zhang, X., Yao, S., Wang, M., Yin, X., Bi, Z., Jing, Y., & Cheng, H. (2022). The Impact of VR‐CALM Intervention Based on VR on Psychological Distress and Symptom Management in Breast Cancer Survivors. *Journal of Oncology*, *2022*(1), 1012813.

Zhao, Q., Liu, B., Sun, Q., & Jin, Y. (2023). Development and validation of a cost‐effective virtual reality educational tool to reduce anxiety and improve set‐up accuracy in radiotherapy patients. *Cancer Medicine*, *12*(5), 6161-6169.

Zhou, Z., Li, J., Wang, H., Luan, Z., Du, S., Wu, N., Chen, Y., & Peng, X. (2023). Experience of using a virtual reality rehabilitation management platform for breast cancer patients: a qualitative study. *Supportive care in cancer*, *31*(5), 307.

Zhou, Z., Li, J., Wang, H., Luan, Z., Li, Y., & Peng, X. (2021). Upper limb rehabilitation system based on virtual reality for breast cancer patients: Development and usability study. *PloS one*, *16*(12), e0261220.
