# Supplementary material for: Extended reality in supporting cancer patients and survivors: A systematic review on the benefits and challenges across the cancer care continuum
Source: Future Healthc J. 2025 Oct 28;13(1):100483. doi: 10.1016/j.fhj.2025.100483 (PMC12769808; doi:10.1016/j.fhj.2025.100483)
Supplement: Supplementary file 2 [file mmc2.docx]

| **Section and Topic** | **Item #** | **Checklist Item** | **Reported in Article** |
| --- | --- | --- | --- |
| **TITLE** | 1 | Identify the report as a systematic review. | Yes (Title, Abstract) |
| **ABSTRACT** | 2 | Structured summary of background, objectives, methods (including data sources), results, discussion, and funding. | Yes |
| **INTRODUCTION** | 3 | Rationale for the review in context of what is already known. | Yes |
|  | 4 | Explicit statement of objectives/questions. | Yes |
| **METHODS** | 5 | Eligibility criteria for inclusion. | Yes (Selection criteria section) |
|  | 6 | Information sources used. | Yes (Databases listed) |
|  | 7 | Full search strategy. | Yes (Described; detailed in Appendix 1) |
|  | 8 | Selection process (number of reviewers, consensus method). | Yes |
|  | 9 | Data collection process (methods and tools used). | Yes |
|  | 10a | Data items sought (e.g., outcomes, interventions). | Yes |
|  | 10b | Assumptions/simplifications made during data collection. | Partial (Some synthesis notes) |
|  | 11 | Risk of bias assessment methods. | Yes (MMAT used) |
|  | 12 | Effect measures for synthesis. | Not Applicable (Mostly narrative synthesis) |
|  | 13a–f | Synthesis methods (e.g., how studies were grouped, heterogeneity assessed, sensitivity analysis). | Yes (Narrative synthesis only; grouping by intervention type and care phase) |
|  | 14 | Reporting bias assessment methods. | Not reported |
|  | 15 | Certainty assessment methods (e.g., GRADE). | Not reported |
| **RESULTS** | 16a–b | Study selection: PRISMA flow diagram and explanation. | Yes (Figure 1 and text) |
|  | 17 | Characteristics of included studies. | Yes (Table 1, narrative description) |
|  | 18 | Risk of bias results. | Yes (Appendix 2 summarized MMAT grades) |
|  | 19 | Results of individual studies. | Yes (Narrative with references) |
|  | 20a–d | Results of syntheses. | Yes (Narrative synthesis and grouped themes) |
|  | 21 | Reporting biases. | Not reported |
|  | 22 | Certainty of evidence. | Not reported |
| **DISCUSSION** | 23a | Summary of findings in relation to objectives. | Yes |
|  | 23b | Limitations of included evidence. | Yes |
|  | 23c | Limitations of the review process. | Yes |
|  | 23d | Implications for practice, policy, and research. | Yes |
| **OTHER INFORMATION** | 24a–c | Registration and protocol information. | Yes (OSF link provided) |
|  | 25 | Support/funding sources. | Not clearly reported |
|  | 26 | Competing interests. | Not reported |
|  | 27 | Availability of data, code, and materials. | Not reported |
